# Reduction Pathway-Dependent Formation of Reactive Fe(II) Sites in Clay Minerals

**DOI:** 10.1021/acs.est.3c01655

**Published:** 2023-07-07

**Authors:** Katherine A. Rothwell, Martin P. Pentrak, Linda A. Pentrak, Joseph W. Stucki, Anke Neumann

**Affiliations:** † School of Engineering, Newcastle University, Cassie Building, Newcastle upon Tyne NE1 7RU, United Kingdom; ‡ Illinois State Geological Survey, Prairie Research Institute, University of Illinois at Urbana-Champaign, Champaign, Illinois 61820, United States; § Department of Natural Resources & Environmental Sciences, University of Illinois at Urbana-Champaign, Urbana, Illinois 61801, United States; △ GFZ German Research Centre for Geosciences, Interface Geochemistry, 14473 Potsdam, Germany

**Keywords:** iron, redox, nontronite, contaminant reduction, kinetics, reactive precipitates, nitroaromatic compounds, Mössbauer spectroscopy

## Abstract

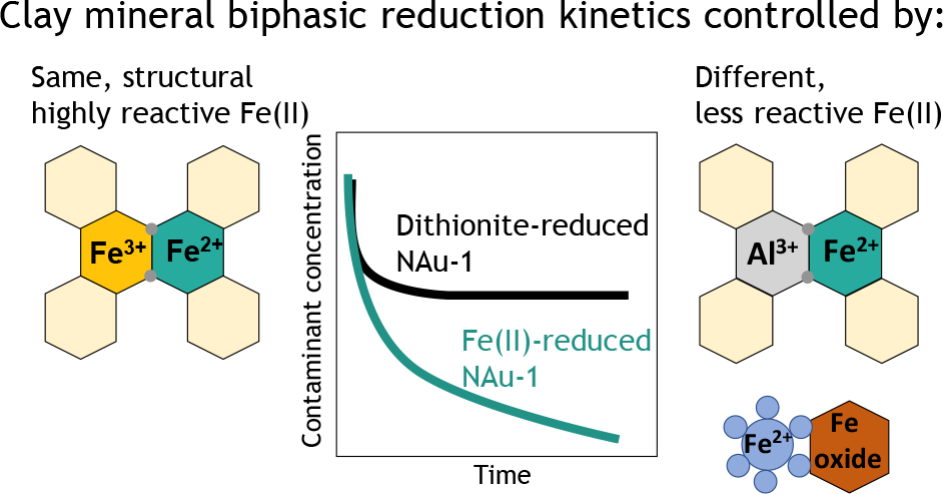

Structural Fe in clay minerals is an important, potentially renewable source of electron equivalents for contaminant reduction, yet our knowledge of how clay mineral Fe reduction pathways and Fe reduction extent affect clay mineral Fe(II) reactivity is limited. Here, we used a nitroaromatic compound (NAC) as a reactive probe molecule to assess the reactivity of chemically reduced (dithionite) and Fe(II)-reduced nontronite across a range of reduction extents. We observed biphasic transformation kinetics for all nontronite reduction extents of ≥5% Fe(II)/Fe(total) regardless of the reduction pathway, indicating that two Fe(II) sites of different reactivities form in nontronite at environmentally relevant reduction extents. At even lower reduction extents, Fe(II)-reduced nontronite completely reduced the NAC whereas dithionite-reduced nontronite could not. Our ^57^Fe Mössbauer spectroscopy, ultraviolet–visible spectroscopy, and kinetic modeling results suggest that the highly reactive Fe(II) entities likely comprise di/trioctahedral Fe(II) domains in the nontronite structure regardless of the reduction mechanism. However, the second Fe(II) species, of lower reactivity, varies and for Fe(II)-reacted NAu-1 likely comprises Fe(II) associated with an Fe-bearing precipitate formed during electron transfer from aqueous to nontronite Fe. Both our observation of biphasic reduction kinetics and the nonlinear relationship of rate constant and clay mineral reduction potential *E*_H_ have major implications for contaminant fate and remediation.

## Abstract 2

This study provides novel kinetic and spectroscopic data on how the pathway of clay mineral Fe reduction affects the resulting redox reactivity, which has significant implications for contaminant transformation in subsurface environments.

## Introduction

Iron-bearing clay minerals are abundant in soils and sediments, and their structurally bound Fe(III) may be reduced via chemical or microbial mechanisms. The resulting structural Fe(II) is capable of reducing a range of contaminants, including nitroaromatic compounds,^1−3^ halogenated hydrocarbons,^4^ pesticides,^5,6^ nitrate,^7^ radionuclides,^8^ and hexavalent chromium.^9,10^ Due to their silicate framework, Fe-bearing clay minerals are largely resistant to reductive dissolution and can thus undergo redox cycling,^11−13^ making them a potentially renewable source of electron equivalents in natural environments.

Results from studies using chemically or microbially reduced Fe-bearing clay minerals suggest that the Fe reduction pathway may control the reactivity of the resulting clay mineral Fe(II). Iron-rich clay mineral nontronite reduced using dithionite (S_2_O_4_^2–^) to >90% Fe(II)/Fe(total) reacted more quickly with nitroaromatic compounds than the same nontronite that had been microbially reduced.^1,3,14^ Moreover, contaminant degradation by Fe-rich clay minerals with high reduction extents, i.e., high Fe(II)/Fe(total) ratios, exhibited characteristic biphasic reaction kinetics that were attributed to the presence of two types of reactive Fe(II) sites within the clay mineral structure.^1,4^ In contrast, organic contaminant transformation by microbially reduced clay minerals followed slower, second-order kinetics, suggesting the presence of only one type of reactive Fe(II) site.^3,15−17^

However, microbial Fe reduction also resulted in much lower reduction extents of ≤40% Fe(II)/Fe(total),^3,11,18^ indicating that the clay mineral Fe reduction extent may exert additional control over the resulting clay mineral Fe redox reactivity. Indeed, in soils, sediments and groundwater, redox potentials typically range from −0.4 to 0.8 V at circumneutral pH,^19^ and hence, Fe reduction extents of ≤40% are expected for Fe-rich clay minerals.^20^ However, it is currently unknown whether chemically reduced Fe-rich clay minerals with lower, and thus more environmentally relevant, Fe reduction extents will still exhibit the same biphasic reaction kinetics as documented for completely reduced clay minerals or display reaction kinetics similar to those of microbially reduced clay minerals.

Additionally, recent work has demonstrated that aqueous Fe(II), which is an abundant reductant in reducing soils, sediments and groundwater, is also capable of reducing clay mineral Fe.^21−24^ Interestingly, this redox reaction results in the formation of an Fe-bearing precipitate(s), the composition of which differs depending on the type of clay mineral, aqueous Fe(II) concentration, pH and solution chemistry,^21−23,25−29^ and that may also be reactive toward contaminants.^24,27,29^ Evidence for how these mineral precipitates affect clay mineral reactivity is sparse and requires a systematic investigation and comparison with reduced clay minerals in the absence of the precipitate(s).

Here, we used a nitroaromatic compound (NAC, 2-acetylnitrobenzene) as a reactive probe to assess the reactivity of the Fe-rich clay mineral, nontronite NAu-1. First, we evaluated the reactivity of dithionite-reduced NAu-1 and systematically varied its reduction extent from 3.5% to 91% Fe(II)/Fe(total), to determine whether biphasic reduction kinetics occur at all reduction extents. Second, to establish similarities and differences with respect to the reactivity of dithionite-reduced NAu-1 and to assess the influence of the Fe precipitate(s), we investigated the reactivity of Fe(II)-reacted NAu-1. We complemented our kinetic assessment with spectroscopic analyses [ultraviolet–visible (UV–vis) and Mössbauer spectroscopy] to identify the reactive Fe(II) species in dithionite- and Fe(II)-reduced NAu-1 and to assess the formation of the mineral precipitate(s).

## Materials and Methods

### Mineral Preparation

A complete list of chemicals used is provided in the Supporting Information (section S1). Nontronite NAu-1 (M_1.05_^+^[Si_6.98_Al_1.02_][Al_0.29_Fe_3.68_Mg_0.04_]O_20_OH_4_;^30^ 21.5 wt % Fe, 100% Fe(III) as measured via HF digestion as described in the next section), was purchased from the Source Clays Repository of The Clay Minerals Society (http://www.clays.org) and size fractionated and Na^+^-homoionized to obtain the 0.1–0.5 μm fraction^31^ (details in section S2). The absence of admixtures was confirmed using Fourier-transform infrared (FT-IR) spectroscopy, and the purified mineral was freeze-dried, gently milled by hand and passed through a 150 μm sieve prior to use.

### Reduction of Fe-Bearing Clay Minerals Using Dithionite

All further mineral manipulation was undertaken in an anaerobic chamber (Glovebox Systemtechnik GmbH) with a N_2_ atmosphere (≤1 ppm O_2_). A modified citrate–bicarbonate–dithionite method was used to reduce the clay mineral Fe.^32,33^ To achieve the desired Fe(II)/Fe(total) ratio, the amount of sodium dithionite salt added was varied according to the stoichiometry required, or for the case of complete reduction, an excess of 3 times the mass of the clay mineral was used. Subsequently, the suspensions were homoionized with Na^+^ and washed with deoxygenated ultrapure water (UPW, ρ ≥ 18.2 MΩ cm). The final concentration of the clay mineral within the stock suspensions (target: 15 g L^–1^) as well as the reduction extent was confirmed by determining the Fe(II) and Fe(total) concentrations, following digestion with hydrofluoric acid (HF), based on a modified 1,10-phenanthroline method.^34,35^

### Reduction Using Aqueous Fe(II)

Solutions of Fe(II) were prepared inside the glovebox by dissolving metallic Fe [Fe(0)] in 1 M HCl at 60 °C followed by dilution with deoxygenated UPW and filtration through a 0.2 μm nylon filter. In addition to Fe(0) in its natural isotopic composition, Fe(II) solutions were also prepared from metallic Fe enriched in the ^56^Fe or ^57^Fe isotope (Isoflex, purity of 99.92% ^56^Fe/∑ ^*i*^Fe or 95.02% ^57^Fe/∑ ^*i*^Fe, respectively).

Batch reactors were prepared in glass vials (20 mL) and contained 15 mL of a 0.5–3.5 mM Fe(II) solution, depending on the desired reduction extent; 10 mM MOPS [3-(*N*-morpholino)propanesulfonic acid] buffer adjusted to pH 7.5 ± 0.1; and 50 mM NaCl as ionic strength buffer. The initial aqueous Fe(II) and Fe(total) concentrations were determined using the colorimetric 1,10-phenanthroline assay.^35^

Powder NAu-1 samples (30.0 ± 0.5 mg) were stored in the glovebox overnight to ensure the absence of oxygen at the mineral surface and were then added to the batch reactors to initiate NAu-1 Fe reduction by aqueous Fe(II). Equilibration was carried out for exactly 24 h on an end-over-end rotator in the dark to prevent photooxidation, to allow the same reduction extent to be reached in all replicate reactors and to prevent aging effects.^2,29^ Then, an aqueous sample was withdrawn, filtered (0.2 μm, nylon) and analyzed for Fe(II) and Fe(total) concentrations (1,10-phenanthroline assay^35^), and the reactors were used for subsequent kinetic experiments.

To quantify the NAu-1 reduction extent and to characterize the solid Fe oxidation product, reactors of the same composition were prepared using ^56^Fe(II) or ^57^Fe(II) solutions, respectively. As demonstrated previously, adding Mössbauer-invisible ^56^Fe(II) allows changes in clay mineral Fe speciation to be monitored,^21,22,26,36^ whereas using Mössbauer-visible ^57^Fe(II) allows the selective assessment of the fate of the added aqueous Fe(II),^21,22,26,36^ as the ^57^Fe in the clay mineral only contributes ≤0.1% of the spectral area in the resulting Mössbauer spectra.

### Kinetic Batch Experiments

Kinetic batch reactors contained Fe(II)-reduced NAu-1 (see above) or dithionite-reduced NAu-1. The latter reactors were prepared from the NAu-1 stock suspension (final concentration: 2 g L^–1^) in a total volume of 15 mL, using 10 mM anoxic MOPS buffer adjusted to pH 7.5 ± 0.1. Reactors contained suspensions of the clay mineral reduced to reduction extents of 3.5–91% Fe(II)/Fe(total) for dithionite-reduced NAu-1 and 3.5–8% Fe(II)/Fe(total) for Fe(II)-reduced NAu-1 (details in Table S2), as confirmed by both Mössbauer spectroscopy and HF digestion with the 1,10-phenanthroline assay.^34,35^ All experiments were performed in triplicate.

Kinetic experiments were initiated by spiking the batch reactors with a methanolic stock solution of 2-acetylnitrobenzene (2AcNB) to give an initial concentration of ∼50 μM. 2AcNB is a nonplanar and hence nonsorbing nitroaromatic compound that has been used widely as a kinetic probe compound in other studies.^1,2,37,38^ Samples (500 μL) were withdrawn periodically, filtered (0.22 μm, nylon) to stop the reaction and stored in the fridge at 4 °C until high-performance liquid chromatography (HPLC) analysis.

### Analytical Method

Quantification of 2AcNB and reduction product 2-acetylaniline (2AcAn) was carried out by HPLC equipped with a diode array detector (Agilent 1260 Infinity II or Thermo Fisher Dionex UltiMate 3000), using an LC-18 column (XBridge C18 3.5 μm) and MeOH/H_2_O (40/60), as previously described.^1^

### UV–Vis Spectroscopy

Quantification of the abundance of Fe(II)–O–Fe(III) species in dithionite-reduced NAu-1 was undertaken using UV–vis spectroscopy (Varian Cary 5) at a wavelength of 730 nm, operating in transmission mode with an integrating sphere detector to minimize signal loss due to light scattering from the clay mineral particles. The spectrometer utilized a flow cell, in which anaerobic conditions were maintained by constant flushing with N_2_.

### Mössbauer Spectroscopy

The structural coordination and reduction extent of Fe in nontronite NAu-1 were analyzed using cryogenic (4–77 K) ^57^Fe Mössbauer spectroscopy. Sample preparation and details of instrument setup and measurements are described in section S3. Spectral fitting was undertaken using the software Recoil (Ottawa, ON)^39^ using a Voigt-based fitting approach^40^ for samples that did not exhibit any magnetic ordering or using Full Static Hamiltonian site analysis for samples containing ordered phases.^41^

### Kinetic Analysis

Kinetics of 2AcNB reduction in nontronite suspensions were fit by applying a second-order kinetic rate law. As previously suggested for Fe-rich clay minerals, the kinetic rate law included the presence of two reactive Fe(II) sites with distinct reactivities (eq 1):^1^
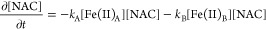
1where second-order rate constants *k*_A_ and *k*_B_ describe the intrinsic reactivities of the Fe(II) sites of high and low reactivity, respectively, [NAC] is the aqueous concentration of 2AcNB and [Fe(II)_A_] and [Fe(II)_B_] are the concentrations of the two reactive Fe(II) sites. We assume that all Fe(II) is redox active and include a mass balance equation for the total Fe(II) concentration, [Fe(II)_total_], in the system as a boundary condition:
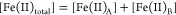
2

For comparison, the kinetic data were also fit with a kinetic rate law including only one reactive Fe(II) site, which results in a simplified version of eq 1 with *k*_B_ set to 0 and the value of [Fe(II)_A_] equal to [Fe(II)_total_]:
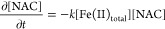
3

Using a least-squares method implemented in Matlab, the appropriate differential equation was used to fit the measured data based on the Nelder–Mead simplex method and resulted in estimated values for *k*_A_, *k*_B_ and the initial concentration of Fe(II)_A_ (eq 1), or *k* (eq 3). Standard deviations of the estimated log parameters were calculated using linear error propagation.

## Results and Discussion

### Effect of Clay Mineral Fe Reduction Extent on Contaminant Reduction

To assess how the Fe reduction extent of Fe-rich clay minerals affects their reaction with contaminants, we monitored the reductive transformation of our reactive probe 2-acetylnitrobenzene (2AcNB)^42^ in suspensions of NAu-1 reduced with dithionite to structural Fe(II)/Fe(total) ratios between 3.5% and 91%. The reactors thus contained 0.32–8.2 mM clay mineral Fe(II) (Table S2), which provides more than the 300 μM electrons required for the stoichiometric transformation of the 50 μM 2AcNB spike to the corresponding aniline [2-acetylaniline (2AcAn)].^42^

**Figure 1:**
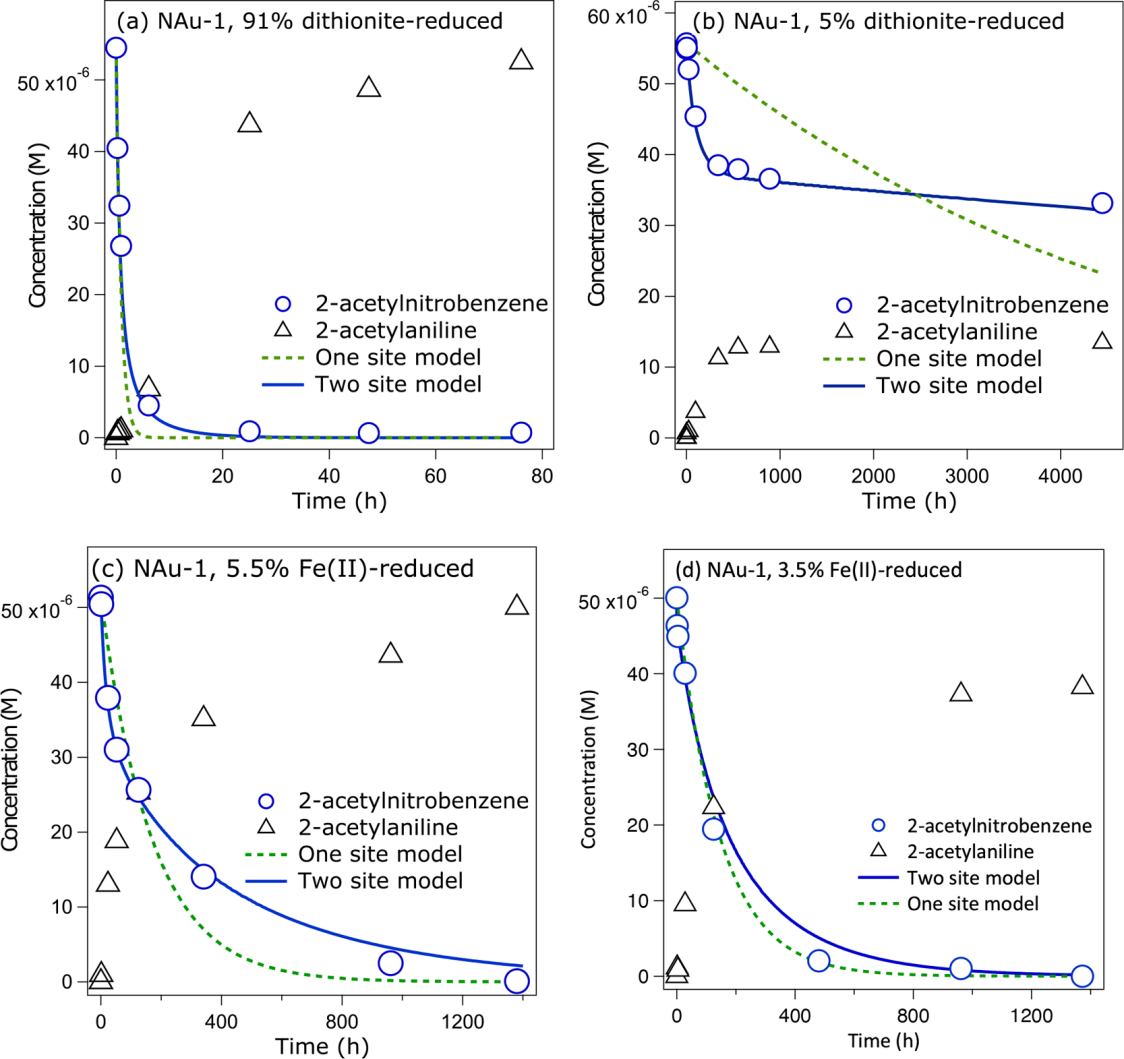
Reduction kinetics of 2-acetylnitrobenzene (blue circles) to 2-acetylaniline (black triangles) in suspensions of (a) 91% dithionite-reduced NAu-1, (b) 5% dithionite-reduced NAu-1, (c) 5.5% Fe(II)-reduced NAu-1, and (d) 3.5% Fe(II)-reduced NAu-1. The solid blue line and dashed green line indicate fits obtained using the two-site (eqs 1 and 2) and one-site kinetic models (eq 3), respectively.

We observed fast NAC reduction kinetics for NAu-1 with reduction extents of 30–91% Fe(II)/Fe(total) (Figure 1a and Figure S1D–F), resulting in complete removal of 2AcNB within <20 h and quantitative formation of 2AcAn in <80 h. For NAu-1 reduction extents of <30% Fe(II)/Fe(total), the reaction became increasingly slower with decreasing NAu-1 Fe reduction extent (Figure S1B,C), and 2AcNB transformation was still incomplete after 4500 h (>6 months) for reactors containing NAu-1 with 5% Fe(II)/Fe(total) (Figure 1b) and negligible for the lowest NAu-1 reduction extent of 3.5% (Figure S1A). Native clay minerals [0% Fe(II)/Fe(total)] have been shown previously to be unable to reductively degrade NACs.^17^ Despite the wide range of 2AcNB transformation rates, the 2AcNB reduction kinetics for all NAu-1 Fe(II)/Fe(total) ratios displayed an initial rapid decrease in 2AcNB concentration, followed by slower reaction. These biphasic transformation kinetics are well represented by our two-site kinetic model (solid blue lines in panels a and b of Figure 1) and could not be fit with a one-site kinetic model (green dashed lines in panels a and b of Figure 1). Previous studies observed similar biphasic reaction kinetics for nitroaromatic compound and halogenated hydrocarbon degradation by a range of Fe-rich smectites that were dithionite-reduced to reduction extents of ≥70%.^1,2,4^ Our results thus confirm these previous observations at high clay mineral Fe reduction extents and, by expanding them to the full range of Fe reduction extents, suggest that biphasic reaction kinetics are an intrinsic property of dithionite-reduced Fe-rich clay minerals independent of their Fe reduction extent.

Because biphasic reduction kinetics are indicative of the presence of two reactive Fe(II) sites in clay minerals,^1,2,4^ our observation of biphasic reduction kinetics for dithionite-reduced NAu-1 across the entire range of Fe(II)/Fe(total) ratios (5–91%) points to the presence of two reactive clay mineral Fe(II) species with different intrinsic reactivities even at very low, and hence environmentally relevant, reduction extents. In contrast, reduction of NACs with microbially reduced nontronite NAu-2, which is similar to NAu-1 in both structure and Fe content, followed a second-order kinetic rate law similar to eq 3.^17^ This kinetic behavior characteristic of the presence of only one reactive Fe(II) site was observed for clay mineral Fe reduction extents of 8–34%,^17^ which coincides with the range investigated in our work. The marked difference in the reaction kinetics of dithionite and biologically reduced clay minerals at similar, low Fe(II)/Fe(total) ratios suggests that the pathway of clay mineral reduction rather than the extent of Fe reduction is critical for the resulting reactivity.

### Nitroaromatic Compound Reduction with Fe(II)-Reduced NAu-1

Our observation that clay mineral reactivity is controlled by the Fe reduction pathway raises the fascinating question of how the reactivity of clay minerals reduced with aqueous Fe(II) will compare to that of microbially reduced and dithionite-reduced clay minerals. We reacted NAu-1 with aqueous Fe(II) concentrations of 0.5–3.5 mM, resulting in the uptake of Fe(II) from solution and subsequent reduction of clay mineral Fe according to a 2:1 stoichiometry (details in section S6). Similar to previous results,^21,23^ we observed a maximum achievable extent of Fe reduction of 8% Fe(II)/Fe(total), despite further increasing aqueous Fe(II) concentrations. Although the maximum reduction extent is much lower than is possible via reduction with dithionite, it is comparable to that observed for microbially reduced NAu-1,^14,43,44^ and the resulting structural Fe(II) concentrations of 0.32–0.72 mM [reduction extents of 3.5–8% (Table S2)] in our kinetic reactors are still in excess to allow stoichiometric transformation of 2AcNB.

Reduction of 2AcNB with Fe(II)-reduced NAu-1 at reduction extents of 5.5% and 8% was characterized by biphasic kinetics (Figure 1c and Figure S2). The similarity in the transformation kinetics of dithionite- and Fe(II)-reduced Fe-rich clay mineral could imply that similar reactive Fe(II) species were formed and/or that the clay mineral Fe reduction pathway is similar for these two abiotic reductants. However, in contrast to dithionite-reduced NAu-1 of comparable reduction extents (Figure 1b), Fe(II)-reduced NAu-1 completely transformed 2AcNB into 2AcAn within 1000 and 1400 h [8% and 5.5% Fe(II)/Fe(total) (Figure 1c)]. Although the same clay mineral Fe(II) concentrations were present in our experiments with Fe(II)- and dithionite-reduced NAu-1, residual aqueous/mineral-associated Fe(II) was present in the reactors after reduction of NAu-1 with Fe(II). Because aqueous Fe(II) alone does not react with 2AcNB (Figure S3),^45,46^ we hypothesize that residual Fe(II) could have interacted with the clay mineral after the initial electron transfer reaction, potentially adding to the reductive capacity of Fe(II)-reduced NAu-1 and/or regenerating the reactive clay mineral Fe(II) via continuous interfacial electron transfer. Alternatively, Fe(II) associated with the potential Fe precipitate(s)^21,26,27,29^ could have contributed to the reactivity, as discussed below.

To explore whether the reduction equivalents stored in the residual Fe(II) could account for the observed differences in reduction capacities of Fe(II)- and dithionite-reduced NAu-1, we calculated an electron balance for the reaction with 2AcNB (Table S3). Addition of NAu-1 to Fe(II) solution removed 0.53–1.47 mM Fe(II) from the aqueous phase, and subsequent electron transfer to the clay mineral Fe, as quantified by Mössbauer spectroscopy, oxidized 0.32–0.72 mM of this solid-associated Fe(II), resulting in 0.21–0.75 mM Fe(II) remaining. Both structural Fe(II) in NAu-1 (0.32–0.72 mM) and solid-associated Fe(II) could each account for a high proportion, if not all, of the 300 μM electrons required for the stoichiometric transformation of the initially added 50 μM 2AcNB observed (Table S3). After 2AcNB reduction, clay mineral Fe(II) had decreased to 0.2–4.7% of the total Fe, indicating that 0.2–0.3 mM reduction equivalents had been transferred from the clay mineral Fe(II), presumably reducing 33–50 μM 2AcNB. Additionally, a further 0.24–0.78 mM Fe(II) had been removed from the aqueous phase, which, together with the solid-associated Fe(II) present before the addition of 2AcNB, could have provided reduction equivalents for the transformation of 77–315 μM 2AcNB. Although our data and mass balance calculations alone do not allow us to unambiguously assign the source of reduction equivalents for 2AcNB transformation, they clearly demonstrate that structural Fe(II) in Fe(II)-reduced NAu-1 became more oxidized during the reaction, and hence contributed to 2AcNB reduction, and that the residual aqueous Fe(II) was redistributed over the course of our experiments.

Interestingly, NAu-1 with a very low reduction extent of 3.5% Fe(II)/Fe(total) transformed 2AcNB stoichiometrically in <1400 h for Fe(II)-reduced NAu-1 (Figure 1d), whereas negligible 2AcNB transformation was observed over 600 h for dithionite-reduced NAu-1 of the same reduction extent (Figure S1A). Moreover, 2AcNB reduction kinetics in the presence of Fe(II)-reduced NAu-1 with a reduction extent of 3.5% were more appropriately described by a second-order kinetic rate law (eq 3, Figure 1d; see section S5 for more details), indicating the presence of only one type of reactive Fe(II) site. Because neither aqueous Fe(II) (Figure S3) nor clay mineral Fe(II) alone (Figure S1A) were capable of reducing 2AcNB, our results suggest that solid-associated Fe(II) formed from the interaction of aqueous Fe(II) with NAu-1, possibly in the form of a mineral precipitate, was responsible for 2AcNB transformation. However, clay mineral Fe(II) became oxidized during the interaction with 2AcNB (Table S3) and might have acted as a reductant for surface-bound Fe, and therefore as a bulk reductant for 2AcNB, with the solid-associated Fe(II) controlling electron transfer kinetics.

### Quantitative Assessment of Clay Mineral Fe(II) Reactivity

To quantitatively compare the reactivity of dithionite- and Fe(II)-reduced NAu-1, we examined the values of rate constants (*k*_A_ and *k*_B_) and the initial concentration of the highly reactive Fe(II) species {[Fe(II)_A_]} resulting from fitting the 2AcNB transformation data with the kinetic models described in eqs 1–3.

**Figure 2:**
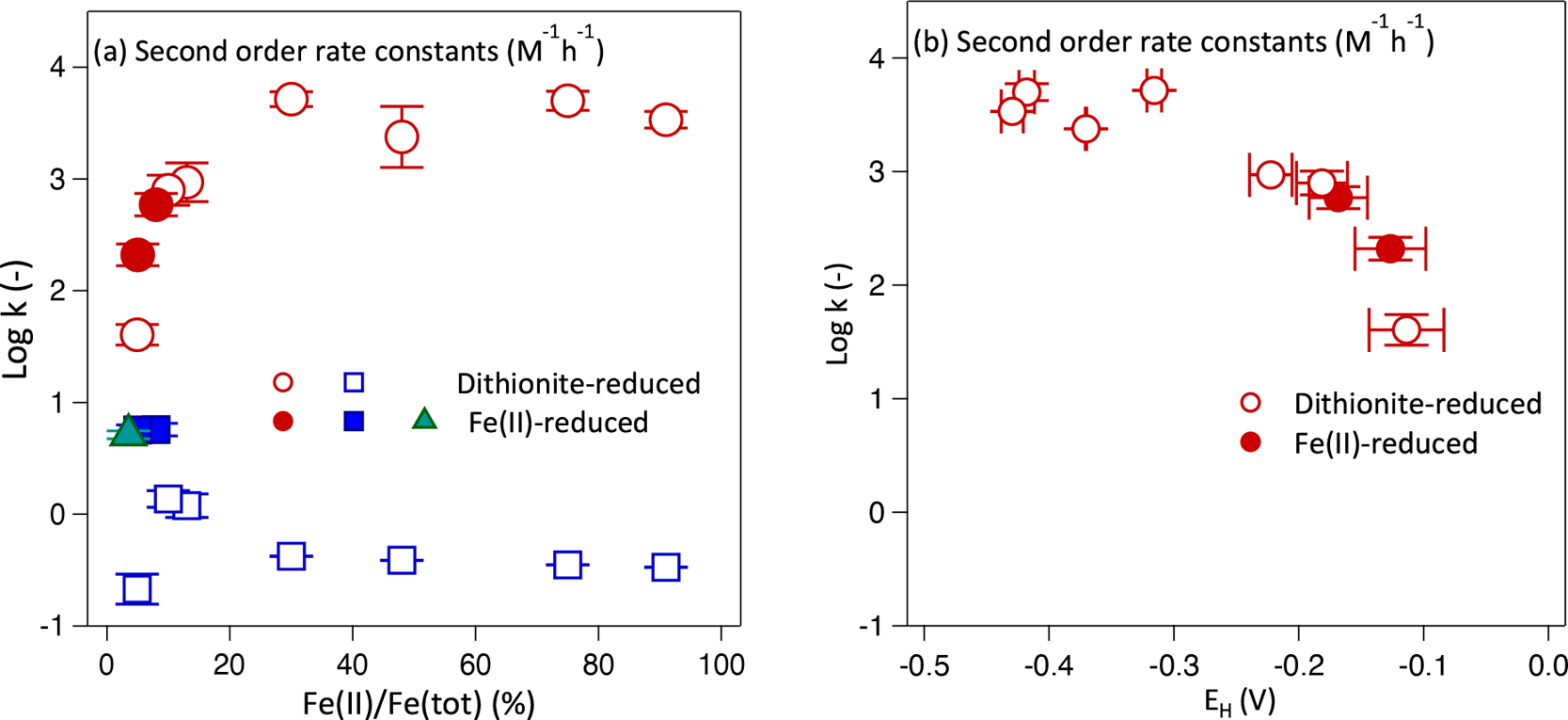
Rate constants (log values) of 2-acetylnitrobenzene (2AcNB) reduction by dithionite-reduced NAu-1 (empty symbols) and Fe(II)-reduced NAu-1 (filled symbols), plotted as a function of (a) NAu-1 reduction extent and (b) NAu-1 Fe(II) reduction potential calculated using the modified Nernst equation and parameters.^49^ Rate constants of highly reactive Fe(II) sites (*k*_A_) are shown as red circles, and those for less reactive Fe(II) sites (*k*_B_) are shown as blue squares. One rate constant was sufficient to describe 2AcNB reduction by 3.5% Fe(II)-reduced NAu-1 and is shown as a green triangle. Error bars indicate the standard deviations of the log values.

Values of rate constant *k*_A_ obtained for dithionite-reduced NAu-1 (red empty circles in Figure 2a) increased by 2 orders of magnitude with an increase in the Fe reduction extent from 5% to 30% Fe(II)/Fe(total) and then plateaued, despite further increases in the Fe reduction extent. As the rate constant is independent of clay mineral Fe(II) concentration (eq 1), this suggests that either different reactive Fe(II) species were involved in probe compound transformation or the intrinsic reactivity of the same Fe(II) species changed with an increase in reduction extent. Although we only have two data points, interestingly, the same trend in *k*_A_ values was observed for Fe(II)-reduced NAu-1 (red filled circles in Figure 2a), albeit over a smaller range of reduction extents and below the threshold at which the rate constant plateaued for dithionite-reduced NAu-1. Thus, we hypothesize that the same reactive Fe(II) species was responsible for the initial fast phase of the reaction or, alternatively, that the same changes in Fe(II) site reactivity occurred, regardless of the clay mineral Fe reduction method applied.

To explore whether the systematic changes in rate constant values were connected to changes in the system’s overall free energy, we investigated the relationship between rate constant *k*_A_ and the clay mineral Fe reduction potential *E*_H_, which we calculated using the modified Nernst equation and redox parameters (*E*_H_^⌀^ and β) for native NAu-1^47^ and the clay mineral Fe reduction extents measured in our reactors (Table S2). We found a steep, nonlinear increase in rate constant values with a decrease in reduction potential between −0.3 and −0.56 V (Figure 2b), suggesting that the clay mineral Fe reduction potential was a controlling factor of the redox reactivity of clay minerals with low Fe reduction extents. A further decrease in reduction potential, corresponding to an increase in clay mineral Fe reduction extent beyond 30% (Table S2), did not lead to higher reduction rate constants, irrespective of whether we used the redox parameters for native or reduced NAu-1 to calculate *E*_H_ (Table S2, Figure 2b, and Figure S7). We thus conclude that for both dithionite- and Fe(II)-reduced NAu-1, a simple (linear) free energy relationship cannot be derived for the entire range of reduction extents and that at a reduction extent of ∼30% Fe(II)/Fe(total), a significant change in the rate-limiting process and/or relative contribution of such processes, for example, mass transfer versus interfacial electron transfer, to the overall transformation occurred. Aeppli et al. recently found that with increasing thermodynamic driving force (*E*_h_) in the system, reduction rates of hematite and goethite converged toward a maximum value that was likely limited by electron transfer into the bulk crystal lattices.^48^ Although our study concerns electron transfer out of the clay mineral lattice, it is conceivable that our observation of an apparent maximum rate constant is caused by a similar limiting process.

Compared to the highly reactive Fe(II) sites, the rate constants of the second, less reactive Fe(II) sites (*k*_B_) were 2–4 orders of magnitude lower in dithionite-reduced NAu-1 (blue empty squares in Figure 2a, and see Table S2), in agreement with previously observed differences between *k*_A_ and *k*_B_ values determined for dithionite-reduced Fe-rich smectites.^1,4,50^ Although the values of *k*_B_ increased slightly with clay mineral Fe reduction extent initially (Figure 2a), log *k*_B_ values remained at the same intermediate level of around −0.5 (Table S2) at reduction extents of ≥30% Fe(II)/Fe(total) and varied by <1 order of magnitude across the entire range of reduction extents.

In contrast to similar trends in *k*_A_ values observed for dithionite- and Fe(II)-reduced NAu-1, the values of *k*_B_ determined for Fe(II)-reduced NAu-1 are notably higher than those of dithionite-reduced NAu-1 (compare blue filled and empty squares in Figure 2a, and see Table S2). Our finding suggests that in Fe(II)- and dithionite-reduced NAu-1, different low-reactivity Fe(II) species were contributing to 2AcNB transformation or that the same Fe(II) species were present but had different reactivities. Interestingly, the value of the rate constant determined for Fe(II)-reduced NAu-1 with a very low Fe(II)/Fe(total) ratio of 3.5% (*k*, triangle in Figure 2a, and see Table S2), where only one reactive site controlled the reaction, coincided with the *k*_B_ values obtained for Fe(II)-reduced NAu-1 with 5.5% and 8% Fe(II)/Fe(total) ratios. Because the same rate constant value [log *k* or log *k*_B_ of 0.71–0.76 (Table S2)] was consistently found for all Fe(II)-reduced NAu-1 in our study, we hypothesize that *k* and *k*_B_ indeed represent the reactivity of the same Fe(II) species in Fe(II)-reduced NAu-1 and that this species is distinct from the low-reactivity Fe(II) site in dithionite-reduced NAu-1. As the dithionite-reduced NAu-1 with 3.5% Fe reduction extent was nonreactive toward the NAC, we ruled out clay mineral structural Fe(II) as the reactive species in Fe(II)-reacted NAu-1 with the same Fe(II)/Fe(total) ratio. Therefore, it is plausible that the low/only reactive Fe(II) species in Fe(II)-reduced NAu-1 could be Fe(II) bound to and/or in the Fe precipitate(s). Published rate constants for nitroaromatic compound transformation with Fe(II)-reacted Fe (oxyhydr)oxides under similar reaction conditions^45,46,51,52^ are of the same order of magnitude as our log *k* and log *k*_B_ values (Table S8) and seem to support this hypothesis, yet unambiguous assignment of the Fe(II) sites in the kinetic model to physical Fe(II) species requires further direct evidence.

In addition to rate constants, the kinetic model also yields the initial concentration of the highly reactive Fe(II) sites {[Fe(II)_A_] in eq 1}. We found almost identical concentrations of Fe(II)_A_ in dithionite- and Fe(II)-reduced NAu-1 (Figure S9), which further corroborates our conclusion that the same highly reactive Fe(II) species were present, irrespective of the clay mineral Fe reduction pathway. Interestingly, [Fe(II)_A_] correlated with the clay mineral reduction extent, and the highly reactive Fe(II) sites became more abundant with an increasing structural Fe(II)/Fe(total) ratio up to a maximum at 48% reduction extent, before decreasing again with further increased reduction extent (Figure S9). The observed trend is similar to that reported for the abundance of octahedral mixed valence Fe(II)–Fe(III) pairs in dithionite-reduced Fe-rich smectites,^53^ indicating these may comprise the reactive entities. However, on the basis of kinetic analysis alone, we cannot identify the clay mineral Fe(II) species or the contribution of the Fe precipitate(s) to the reactivity of Fe(II)-reacted clay minerals.

### Linking Reactive Fe(II) Sites to Structural Entities in Reduced Nontronite

**Figure 3:**
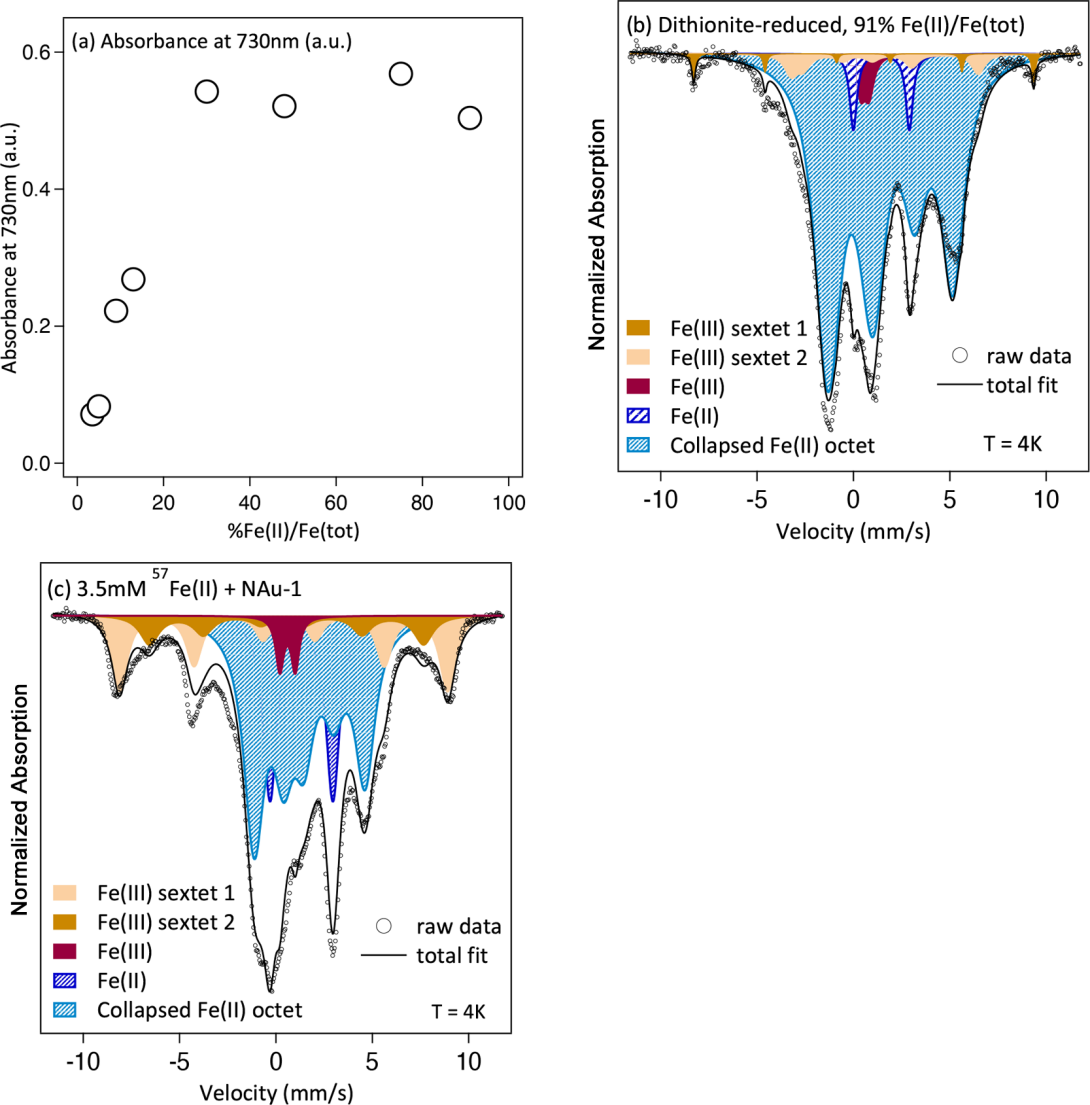
Data from (a) UV–vis and (b and c) Mössbauer spectroscopic measurements on dithionite- and Fe(II)-reduced NAu-1. (a) Absorbance values measured at 730 nm as a function of Fe reduction extent in dithionite-reduced NAu-1 indicating an initial increase in Fe(II)–Fe(III) intervalence charge transfer,^53^ which plateaus at reduction extents exceeding ∼30% Fe(II)/Fe(total). (b) Mössbauer spectrum collected at 4 K of dithionite-reduced NAu-1 with an Fe(II)/Fe(total) ratio of 91% showing the majority of the spectral area comprising an octet with fitting parameters similar to a trioctahedral biotite.^54^ (c) Mössbauer spectrum collected at 4 K of NAu-1 reacted with 3.5 mM Mössbauer-visible ^57^Fe(II) showing the composition of the Fe precipitate (the clay mineral signal is <0.1% of the spectral area).

To link the reactive Fe(II) sites used in the kinetic model to specific Fe entities within the clay mineral structure, we first used the absorbance of the Fe(II)–Fe(III) intervalence charge transfer (IVCT) band at 730 nm as a measure for the abundance of Fe(II)–O–Fe(III) species [i.e., octahedral Fe(II)–Fe(III) pairs] in reduced nontronite.^18,53^ As expected, we observed an increase in absorbance with increasing Fe reduction extent of dithionite-reduced NAu-1 (Figure 3a), suggesting an increasing concentration of Fe(II)–O–Fe(III) entities. However, the absorbance measured at 730 nm remained at similar levels for NAu-1 reduced to Fe(II)/Fe(total) ratios of ≥30% (Figure 3a), indicating a constant amount of Fe(II)–O–Fe(III) entities. This apparent plateau in absorbance clearly contrasts previous observations of a distinct absorbance maximum at nontronite reduction extents of around 40–50%^18,53^ and also differs from the trend observed for the concentration of highly reactive Fe(II) sites (Figure S9) in our kinetic model. Thus, our results from IVCT band measurements seem to suggest that Fe(II)–O–Fe(III) species in NAu-1 are unlikely to correspond to the highly reactive Fe(II) sites.

Alternatively, the interpretation of the absorbance band at 730 nm as corresponding to static Fe(II)–O–Fe(III) entities can be expanded to include recent insights into electron mobility within the nontronite lattice. At room temperature, at which the absorbance measurements were carried out, electron hopping between neighboring Fe atoms in the octahedral sheet of nontronites is fast.^55^ The resulting, at least partial, delocalization of electrons within the clay mineral structure was confirmed for partially reduced nontronites by temperature-dependent observations of distinct Fe(II) entities with Mössbauer spectroscopy^21^ and the absence of a distinct absorption band corresponding to clay mineral mixed valence Fe(II)–Fe(III) pairs in room-temperature infrared (IR) spectra.^50^ Furthermore, we calculated that at low NAu-1 Fe reduction extents [≤15% (section S11)] that coincide with increasing absorbance values measured at 730 nm, the clay mineral dioctahedral structure is preserved, as cation sorption dominates over proton uptake and subsequent rearrangements of the clay mineral structure.^56^ We thus suggest that, at low clay mineral Fe reduction extents, the absorbance band at 730 nm could be an indicator of an increased electron load of the dioctahedral clay mineral structure^57^ rather than linked to the number of distinct Fe(II)–O–Fe(III) entities. This interpretation fits well with our observation of increased intrinsic reactivity of clay mineral Fe(II), i.e., rate constants (Figure 2a), due to increased electron loading rather than increased numbers of highly reactive sites, which remained constant at reduction extents of <20% (Figure S9).

At higher reduction extents, the uptake of protons increasingly dominates over cation sorption (section S11) to balance the charge created in the octahedral sheets by the formation of Fe(II)^56^ and leads to subsequent dehydroxylation and clay mineral structural rearrangements, including trioctahedral domain formation.^56,58^ If the trioctahedral entities comprise almost exclusively Fe(II) atoms and contain appreciable amounts of Fe(III) above the threshold of 1/10 Fe(III)/Fe(II), further Fe reduction and structural rearrangements will not result in a change in absorbance at 730 nm,^59^ suggesting that our observations are consistent with the formation of Fe(II) trioctahedral entities at reduction extents exceeding 30%. In previous work with fully reduced Fe-rich smectites and nontronites, trioctahedral Fe(II) entities have been linked to highly reactive Fe(II) sites in the kinetic model, whereas dioctahedral Fe(II) paired with octahedral cations, including Al and Fe(II), was related to sites with a lower reactivity.^50^ The question of whether this interpretation will hold true for our results from dithionite-reduced NAu-1 with much lower Fe(II)/Fe(total) ratios (30–70%) than those investigated previously remains unanswered.

To explore the extent of Fe(II) domain formation in our dithionite-reduced NAu-1, we used cryogenic Mössbauer spectroscopy at temperatures between 77 and 4 K. Mössbauer spectra collected at 77 K (Figure S10) showed clearly defined Fe(III) and Fe(II) doublets for NAu-1 samples across the complete range of Fe reduction extents (3.5–91%), indicating that electron hopping between adjacent Fe atoms was slower than the Mössbauer characteristic time (10^–8^ s).^21^ When the same samples were cooled to 13 K, NAu-1 reduced to Fe(II)/Fe(total) ratios between 3.5% and 13% retained the same defined Fe(III) and Fe(II) doublets (Figure S10a–d and Table S4), whereas we observed an additional, poorly resolved spectral feature due to partial magnetic ordering in spectra of NAu-1 reduced to Fe(II)/Fe(total) ratios of ≥30% (Figure S10f,g). Partial magnetic ordering occurred at the same NAu-1 reduction extent at which the absorbance at 730 nm plateaued and thus could indicate the onset of Fe clustering in the nontronite structure. a similar increase in magnetic ordering temperature with an increase in Fe(II) content has been observed for a range of clay minerals and has been assigned to an increased Fe(II) ferromagnetic component and increased charge transfer between neighboring Fe(II) and Fe(III) atoms,^53,54^ rather than Fe clustering.

Mössbauer spectra recorded at an even lower temperature of 4 K show that magnetic ordering occurred in all dithionite-reduced NAu-1 samples (Figure S11) and increased with an increase in Fe reduction extent, consistent with an increasing Fe(II) ferromagnetic component in the mineral.^54^ Magnetic ordering was not well developed and evident only as a broad, poorly resolved feature in the Mössbauer spectra of NAu-1 with Fe(II)/Fe(total) ratios of <20% (gray area in Figure S11a). The lack of distinct hyperfine interactions in spectra collected at 4 K has been previously interpreted as a lack of spatially segregated Fe(II) and Fe(III) domains, i.e., the absence of Fe clustering.^14^ Our observations thus suggest the presence of mixed valence Fe(II)–Fe(III) domains in dithionite-reduced NAu-1 with Fe(II)/Fe(total) ratios of <20% and corroborate our conclusion that at these reduction extents NAu-1 contains mostly dioctahedral reactive Fe(II) species.

In contrast, Mössbauer spectra of NAu-1 dithionite-reduced to extents of ≥20% contained distinct magnetically ordered components (blue shaded and brown areas in Figure 3b and Figure S10), similar to those observed for fully dithionite-reduced Garfield nontronite^14^ and indicative of clustering of Fe into spatially segregated Fe(II) and Fe(III) domains. While the Mössbauer spectrum of NAu-1 with an Fe(II)/Fe(total) ratio of 20% (Figure S11B) shows both magnetically ordered Fe(II) and Fe(III) components and the same poorly ordered component (gray area) observed at lower reduction extents, the latter is absent in Mössbauer spectra of NAu-1 with higher Fe reduction extents [≥48% (Figure 3b and Figure S11C)], suggesting that Fe clustering was incomplete in NAu-1 at an Fe reduction extent of 20%. With increasing NAu-1 Fe reduction extent, both the area and the hyperfine magnetic field value [*H* (Table S5)] of the magnetically ordered Fe(II) component (blue shaded area in Figure 3b and Figure S11) increased, indicating increasing levels of Fe(II) clustering in the NAu-1 structure. The Mössbauer spectrum of NAu-1 reduced to 91% Fe(II)/Fe(total) (Figure 3b) was dominated by the Fe(II) octet, which occupied 87% of the spectral area and exhibited hyperfine parameters [*H* = 17.3 T (Table S5)] similar to those reported previously for magnetically ordered Fe(II) in trioctahedral annite and ferrous biotite^54,60^ (Table S6, detailed discussion of the hyperfine parameters in section S13), confirming the dominant presence of trioctahedral Fe(II) domains in nontronites reduced to high extents.

Our combined spectroscopic and kinetic analyses thus suggest that at low reduction extents (≤20%), the highly reactive Fe(II) site in the kinetic model corresponds to dioctahedrally bound Fe(II). This dioctahedral Fe(II) is most likely connected to Fe(III) and exhibits an increasing reactivity (*k*_A_) due to increased electron loading and electron delocalization/mobility within the octahedral sheet, rather than an increase in abundance {[Fe(II)_A_]} with an increase in reduction extent. Because the kinetic parameters of the highly reactive Fe(II) site {*k*_A_ and [Fe(II)_A_]} are identical for dithionite- and Fe(II)-reduced NAu-1, and because Mössbauer spectra recorded at 13 K show no magnetic ordering (Figure S6), we suggest that the same reactive Fe(II) species, i.e., dioctahedral Fe(II), gave rise to the highly reactive Fe(II) site in the kinetic model, regardless of the reductant used. Only at reduction extents exceeding 20% does proton sorption dominate over cation uptake and lead to dehydroxylation and the formation of trioctahedral Fe(II) domains, which then comprise the highly reactive Fe(II) site. We hypothesize that these trioctahedral entities are initially small, minimizing the energy required for concomitant structural rearrangements to take place.^56^ As reduction increases further, increasing the number of small trioctahedral groups becomes energetically less favorable, and instead, the size of the trioctahedral domains increases, decreasing their abundance yet maintaining their intrinsic reactivity (*k*_A_). This hypothesis is consistent with a maximum of highly reactive site abundance {[Fe(II)_A_]} around 50% Fe(II)/Fe(total) in NAu-1 and its subsequent decrease at higher reduction extents (Figure S9).

### Low-Reactivity Fe(II) Sites and Fe Precipitate Formation

In contrast to the similarities in highly reactive Fe(II) sites in Fe(II)- and dithionite-reduced NAu-1, the differences in the rate constants of the low-reactivity sites (Figure 2a) suggest the presence and involvement of different Fe(II) species, dependent on the clay mineral Fe reduction pathway. We used the isotope specificity of ^57^Fe Mössbauer spectroscopy in combination with aqueous Fe(II) enriched within the ^57^Fe isotope to study the fate of the aqueous Fe(II) reacted with NAu-1.^21,26^ Mössbauer spectra recorded at 4 K of ^57^Fe(II)-reacted NAu-1 samples equivalent to those of NAu-1 with Fe(II)/Fe(total) ratios of 3.5–8% all exhibit magnetically ordered components (blue shaded and brown areas in Figure 3c and Figure S12), indicating that, as atom exchange is negligible under our reaction conditions, most ^57^Fe taken up from solution was bound in newly precipitated mineral phases rather than sorbed to clay minerals.^22,25,61^ Most strikingly, we found a large Fe(II) octet component (blue shaded area in Figure 3c), comprising 31–52% of the spectral area (Table S7), in addition to the Fe(III) expected to result after electron transfer from aqueous Fe(II) to clay mineral Fe(III). The hyperfine parameters of these ordered components (Table S7) are consistent with a range of Fe(II)- and Fe(III)-bearing phases, including, but not limited to, ferrihydrite [Fe(III) sextet 1],^61^ lepidocrocite [Fe(III) sextet 2],^61^ and green rust [Fe(II) octet and Fe(III) sextets],^62^ in agreement with previous studies suggesting the formation of ferrihydrite,^27^ green rust,^24,29^ and/or Fe(II)Al(III)-layered double hydroxides^28^ under similar reaction conditions. Irrespective of the specific identity of the secondary Fe precipitate(s), it is plausible that the Fe(II) species corresponding to the Fe(II) octet represents the low-reactivity Fe(II) sites, in analogy to our assignment of the magnetically ordered Fe(II) component in dithionite-reduced clay minerals to the highly reactive Fe(II) site.

However, all Mössbauer spectra also exhibit an Fe(II) doublet (dark blue shaded areas in Figure 3c and Figure S12) comprising substantial spectral areas of 18–20% (Table S7), and this Fe(II) component, due to the absence of magnetic ordering, is likely present as sorbed to a mineral phase.^22,61^ Because Fe(II) sorbed to Fe oxides, hydroxides, silica, alumina, and Fe-free clay minerals can also transform nitroaromatic compounds,^46,51,52^ the sorbed Fe(II) in our system could also correspond to the low-reactivity Fe(II) site in Fe(II)-reduced NAu-1. Comparison with rate constants in the literature (Table S8) suggests that Fe(II) sorbed to an Fe(III) (oxyhydr)oxide like lepidocrocite rather than to silica or alumina surfaces as found in clay minerals will more likely result in similar rate constants (log *k*_B_) as observed here. Although we cannot unambiguously conclude which of the Fe(II) components corresponds to the low-reactivity site in Fe(II)-reduced NAu-1, we can conclude that one solid-bound Fe(II) component or both comprise the low-reactivity site. These components are present in the spectra of all Fe(II)-reacted NAu-1 samples, most importantly in the spectrum of Fe(II)-reacted NAu-1 reduced to only 3.5% Fe(II)/Fe(total), where only one, non-clay mineral reactive site was responsible for 2AcNB transformation.

## Environmental Implications

Our findings significantly expand the understanding of how the mechanism of Fe reduction controls the formation of reactive sites in the structure of Fe-rich clay minerals. Consequently, observations of different redox reactivities observed in experiments can be rationalized with our observation of two reactive Fe(II) sites in abiotically reduced clay minerals [by Fe(II) and dithionite] compared to previous reports of the presence of only one reactive Fe(II) site in microbially reduced clay minerals.^3,15−17^ Importantly, aqueous Fe(II) is ubiquitous in subsurface environments,^63^ making our results directly relevant to the assessment of contaminant degradation and fate. Because our two-site kinetic model that we developed for Fe-rich clay minerals of high Fe reduction extents^1^ could also be applied to clay mineral Fe reduction extents observed in natural subsurface environments (5–30%^49^) and Fe(II)-reduced NAu-1, we suggest that this model should be included in reactive transport models to appropriately quantify contaminant degradation.

Our data set covers a uniquely large range of clay mineral reduction extents [5–91% Fe(II)/Fe(total)], encompassing what has been investigated in previous studies that linked contaminant degradation rate constants to clay mineral Fe reduction potential, *E*_H_, in linear free energy relationships (LFERs).^10,17^ Interestingly, the LFER proposed for the reduction of NAC by microbially reduced nontronite NAu-2 covers low clay mineral Fe reduction extents of 21–34%,^17^ and our rate constants for the structurally highly similar nontronite NAu-1 could also be fit to a linear regression (*R*^2^ = 0.83) within a similar range of clay mineral Fe reduction extents [10–30% (Figure S8)]. Even though the slopes of the LFERs differ [microbially reduced NAu-2, 1.16; dithionite- and Fe(II)-reduced NAu-1, this study, 0.75], this comparison suggests that the redox reactivity of Fe-rich clay minerals of low reduction extents (≤30%) is controlled by the bulk clay mineral reduction potential irrespective of the reduction pathway. This parameter could be easily measured in the field and/or on samples collected from contaminated sites and could be used to predict transformation rates and extents.

However, our data also clearly demonstrate that for nontronite reduction extents of >30% Fe(II)/Fe(total), deviations from the linear behavior (Figure 2b) occur, suggesting that the LFER fails for larger variations in clay mineral *E*_H_. Similar nonlinear characteristics can be observed in the LFER proposed for the reduction of Cr(VI) by dithionite-reduced nontronite NAu-2, and we suggest that the linear relationship proposed might be an oversimplified interpretation of the data collected for clay mineral Fe reduction extents ranging from 26% to 98% Fe(II)/Fe(total).^10^ Moreover, our observation of rate constants plateauing at clay mineral Fe reduction extents of ≥30%, and hence becoming disconnected from further decreasing clay mineral *E*_H_ values, is highly relevant for active contaminant remediation approaches such as reactive barriers or in situ chemical reduction,^64,65^ where high clay mineral Fe reduction extents are plausible and/or dithionite might be used as an in situ reductant.^66^ Here, considerations of both biphasic reduction kinetics and the deviation from the linear relationship of the rate constant and *E*_H_ could be useful for optimizing barrier design and/or reductant dosing.

One of our most intriguing findings, though, is the relative contribution of Fe(II) associated with the Fe-bearing precipitate(s) to contaminant degradation in suspensions of Fe(II)-reduced NAu-1. Even though these low-reactivity sites dominated [≥5% Fe(II)/Fe(total)] or even fully controlled [3.5% Fe(II)/Fe(total)] contaminant reduction kinetics, Fe(II) in NAu-1 also became oxidized and hence acted as the bulk reductant, or redox buffer. This role of clay mineral Fe is highly environmentally significant yet has been largely overlooked in previous investigations of Fe(II)-reduced clay minerals.^27,29^ Our observation of two coexisting Fe-bearing mineral phases exhibiting, at apparent equilibrium, Fe(II) sites with intrinsically different redox reactivities, or rate constants (*k*_A_ and *k*_B_), also enables us to rationalize how Fe sites of different redox reactivities can exist in clay minerals at one reduction potential. Just as the binding environments of Fe(II) in the clay mineral and Fe-bearing precipitates equip these sites with different reactivities, also differently bound Fe(II) in the clay mineral structure could lead to different redox reactivities. In particular, electron transfer from clay mineral Fe(II) results in the reversal of structural rearrangements that occurred during reduction,^14,50^ such that it is conceivable that trioctahedral Fe(II) and dioctahedral Fe(II) could exhibit different observable kinetics of electron transfer.

## Supporting Information Available

Reduction mechanism-dependent formation of reactive Fe(II) sites in clay minerals (PDF)
