# Supplementary material for: Reduction Pathway-Dependent Formation of Reactive Fe(II) Sites in Clay Minerals
Source: Environ Sci Technol. 2023 Jul 7;57(28):10231–41. doi: 10.1021/acs.est.3c01655 (PMC10357582; doi:10.1021/acs.est.3c01655)
Supplement: Supplementary file 1 — es3c01655_si_001.pdf [file es3c01655_si_001.pdf]

Reduction pathway-dependent formation of reactive Fe(II) sites in clay minerals: Supporting Information

Katherine A. Rothwell*[1,4], Martin P. Pentrak[2], Linda A. Pentrak[3], Joseph W. Stucki[3], and Anke Neumann†[1]

[1]School of Engineering, Newcastle University, Cassie Building, Newcastle upon Tyne, NE1 7RU, UK.

[2]Illinois State Geological Survey, Prairie Research Institute, University of Illinois at Urbana-Champaign, 61820, Champaign USA

[3]Department of Natural Resources & Environmental Sciences, University of Illinois at Urbana-Champaign, Urbana, IL 61801, USA

[4]Now at: School of Earth Sciences, Wills Memorial Building, Bristol, BS8 1RJ, United Kingdom

Pages: 27
Figures: 12
Tables: 8

*k.rothwell@bristol.ac.uk
†anke.neumann@ncl.ac.uk

Contents

S1 Chemicals and Reagents S3

S2 Mineral preparation S3

S3 Mössbauer spectroscopy S3

S4 NAC reduction by dithionite-reduced NAu-1 S4

S5 NAC reduction by Fe(II)-reduced NAu-1 S5

S6 Reduction of NAu-1 by aqueous Fe(II) S6

S7 Mössbauer spectra of ^{56}Fe(II)-reduced NAu-1 S7

S8 Input and output parameters of the two-site kinetic model S10

S9 NAu-1 Reduction Potential S11

S10 Electron Balance Calculations S13

S11 Calculation of reduction extent at which proton uptake dominates over cation sorption S15

S12 Mössbauer spectra of dithionite-reduced NAu-1 S16

S13 Mössbauer spectra of dithionite-reduced NAu-1 at 4K S19

S14 Mössbauer parameters for Fe-bearing phyllosilicates from literature S22

S15 Mössbauer spectra of ^{57}Fe(II)-reduced NAu-1 S23

S16 Reactivity of sorbed Fe(II) S25

S1 Chemicals and Reagents

All chemicals and reagents used in this study were of analytical grade and purchased from either Fisher Scientific/ACROS Organics (sodium chloride, sodium citrate, sodium bicarbonate, sodium dithionite, 1,10-phenanthroline, hydrofluoric acid, hydrochloric acid) or Sigma Aldrich (sodium hydroxide, 2-acetylnitrobenzene, 2-acetylaniline, HPLC grade methanol). All stock solutions were purged with N_2 for at least an hour before introduction to the anaerobic chamber.

S2 Mineral preparation

The 0.1-0.5 μm fraction of the clay mineral was obtained through repeated centrifugation according to Stoke's law. The fractionated clay mineral was washed three times in 1 M NaCl solution, in order to achieve Na^+ homoionization and then washed twice with DI water. Finally, mineral impurities were removed by repeated centrifugation for 5 minutes at 8000 rpm, which preferentially leaves the impure fraction in the pellet and the clean fraction in the supernatant. FT-IR spectroscopy was used to confirm the removal of any Fe-oxide, carbonate and kaolin phases.

S3 Mössbauer spectroscopy

Samples were prepared for Mössbauer analyses in the anaerobic chamber by extracting the clay mineral solids from the reactors by centrifugation and then placing a small amount between two pieces of Kapton tape, to prevent exposure to oxygen during transfer to the Mössbauer spectrometer.

Mössbauer spectra were collected in transmission mode using a MS4 Mössbauer spectrometer (SEE Co., Edina, MN, USA) by attaching the sample to a rod using Kapton tape and inserting into a closed cycle helium cryostat (SHI-850, Janis Research Co., Wilmington, MA, USA). Data was calibrated against α-Fe foil.

S4 NAC reduction by dithionite-reduced NAu-1

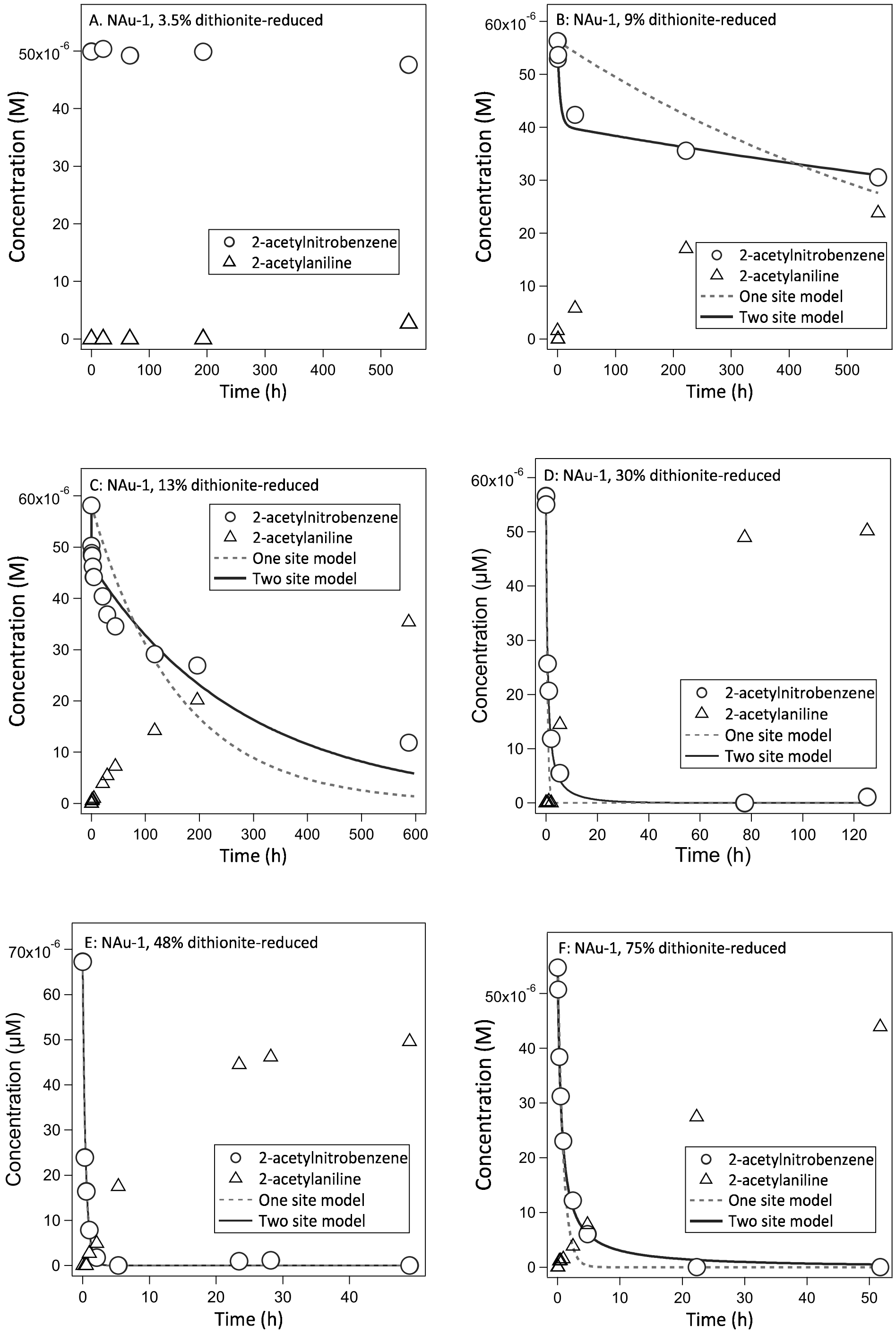

Figure S 1: Typical reduction kinetics of 2-acetylnitrobenzene (blue circles) to 2-acetylaniline (black triangles) in suspensions of dithionite-reduced NAu-1 with initial clay mineral Fe reduction extent of (a) 3.5% Fe(II)/Fe(total) (b) 9% Fe(II)/Fe(total), (c) 13% Fe(II)/Fe(total), (d) 30% Fe(II)/Fe(total), (e) 48% Fe(II)/Fe(total) and (f) 75% Fe(II)/Fe(total). The solid blue line and the dashed green line indicate fits obtained using the two-site (eqs 1-2) and one-site kinetic models (eq 3), respectively.

S5 NAC reduction by Fe(II)-reduced NAu-1

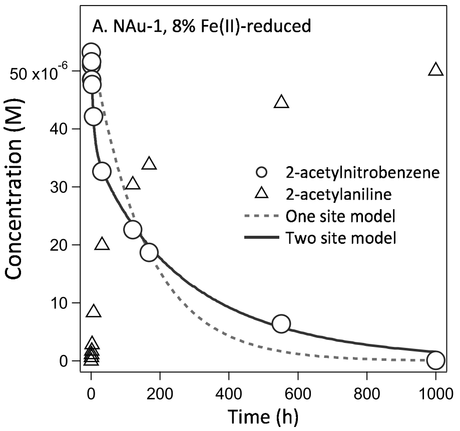

Figure S 2: Reduction kinetics of 2-acetylnitrobenzene (blue circles) to 2-acetylaniline (black triangles) in a suspension of Fe(II)-reduced NAu-1 with a reduction extent of 8% Fe(II)/Fe(total). The solid blue line and the dashed green line indicate fits obtained using the two-site (eqs 1-2) and one-site kinetic models (eq 3), respectively.

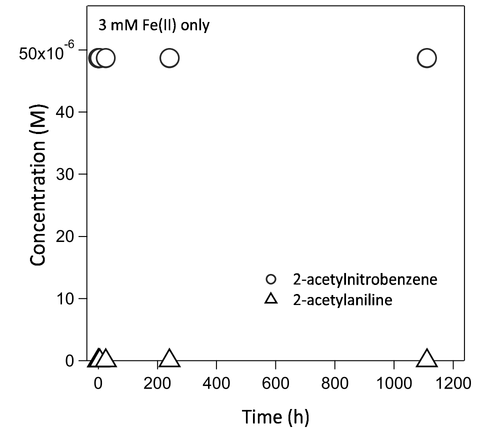

Figure S 3: Concentrations of 2-acetylnitrobenzene (blue circles) and 2-acetylaniline (black triangles) in solution containing 3.0 mM aqueous Fe(II) at pH 7.5 were monitored over more than 1000 hours (>1 month), showing no change or indication of reductive transformation.

We note that, qualitatively, the rate of 2-acetylnitrobenzene (2AcNB) reduction may seem to be lower in the presence of Fe(II)-reduced NAu-1 with 5.5% reduction extent (Fig 1c) compared to 3.5% reduced NAu-1 (Fig 1d), when based on the 2AcNB concentration falling to 0 after shorter reaction time. We attribute this observation to two phenomena: 1) small variations in clay mineral loading/ concentration leading to slight differences in observable rate, and 2) different types of kinetic rate law applicable to each of these two 2AcNB reduction experiments. Both phenomena can be accounted for by deriving the rate constant(s) from the rates of reduction,

as explained in the Methods section (Kinetic Analysis), enabling comparison despite small variations in clay mineral Fe(II) and total Fe content, added aqueous Fe(II) concentration and different types of (second-order) kinetic rate laws.

S6 Reduction of NAu-1 by aqueous Fe(II)

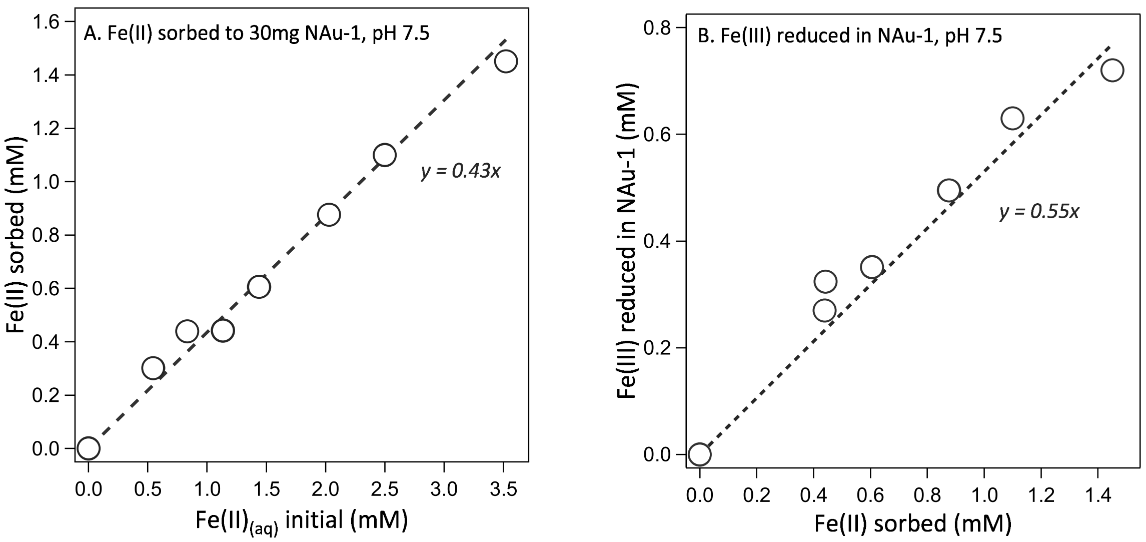

Figure S 4: Stoichiometry of the reaction between sorbed Fe(II) and structural Fe(III) reduced in NAu-1.

A maximum reduction extent of 8% Fe(II)\Fe(tot) was achieved, which is comparable to that observed by [1] for microbially reduced NAu-1 following the addition of electron shuttle AQDS. For particle size 0.1-0.5μm, which we have selected for in our reactors, we calculated that approximately 0.2% - 1.3% of the structural Fe in NAu-1 is located at the edges and therefore the 8% reduction corresponds to internal as well as edge-bound Fe(II), contrary to the suggestion made by [1] that only edge-bound Fe was reduced in NAu-1. Other possibilities that may limit further reduction include charge balance limitations [2], the presence of a solid oxidation product at the mineral surface preventing further sorption and therefore electron transfer from Fe(II) or thermodynamic limitations.

It is unclear why the relationship does not show a 1:1 relationship similar to that observed by [3] for nontronite NAu-2: in NAu-1 only approximately 50% of the sorbed Fe(II) is able to reduce structural Fe. However, Fe in NAu-2 has also been shown to be more microbially available with up to 32% structural Fe reduction observed by [4] using *Shewanella putrefaciens* strain CN32 with AQDS compared to the 8% observed for NAu-1 by [1] with *Shewanella oneidensis* strain MR-1 and AQDS. Furthermore, NAu-2 also contains tetrahedral Fe not present in NAu-1.

S7 Mössbauer spectra of ^{56}Fe(II)-reduced NAu-1

As ^{56}Fe(II) is not visible to Mössbauer Spectroscopy, the following ^{57}Fe Mössbauer Spectra only show the naturally abundant ^{57}Fe in the clay mineral structure, and thus clay mineral reduction extent. These spectra were used to calculate the structural Fe(II) concentration in Tables S2 and S3.

As detailed in Table S1, the spectra generally show an octahedral Fe(III) doublet comprising most of the Fe(III) spectral area (black dots) with parameters centre shift (CS) = 0.47 - 0.49, quadrupole splitting (QS) = 0.35 - 0.50, a second, smaller octahedral Fe(III) doublet (black dashes) with parameters CS = 0.70 - 0.74, QS = 0.08 - 0.18, and an Fe(II) doublet (filled blue) with the parameters CS = 1.20 - 1.22, QS = 2.85 - 3.00.

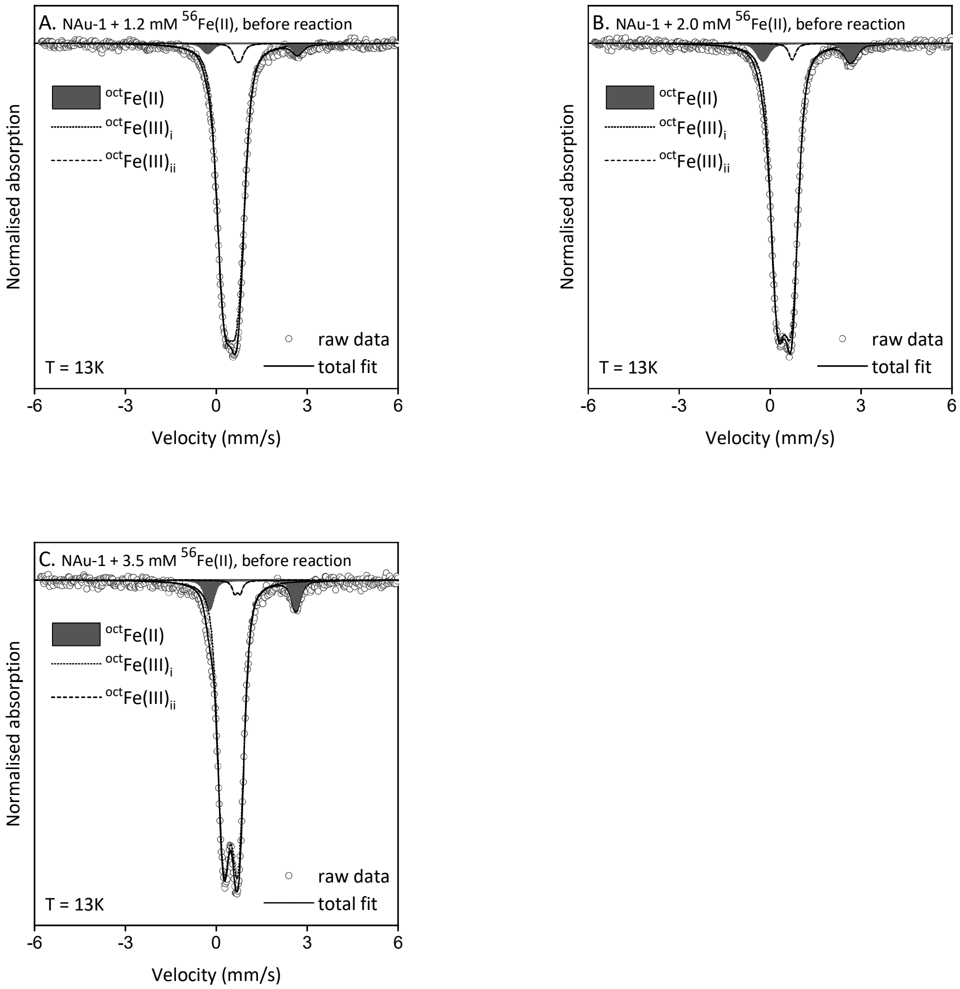

Figure S 5: Mössbauer spectra measured before reaction with 2AcNB of (a) 1.2 mM ^{56}Fe(II) + NAu-1 (3.5 % Fe(II)/Fe(tot)), (b) 2.0 mM ^{56}Fe(II) + NAu-1 (5.5 % Fe(II)/Fe(tot)), and (c) 3.5 mM ^{56}Fe(II) + NAu-1 (8.0 % Fe(II)/Fe(tot)).

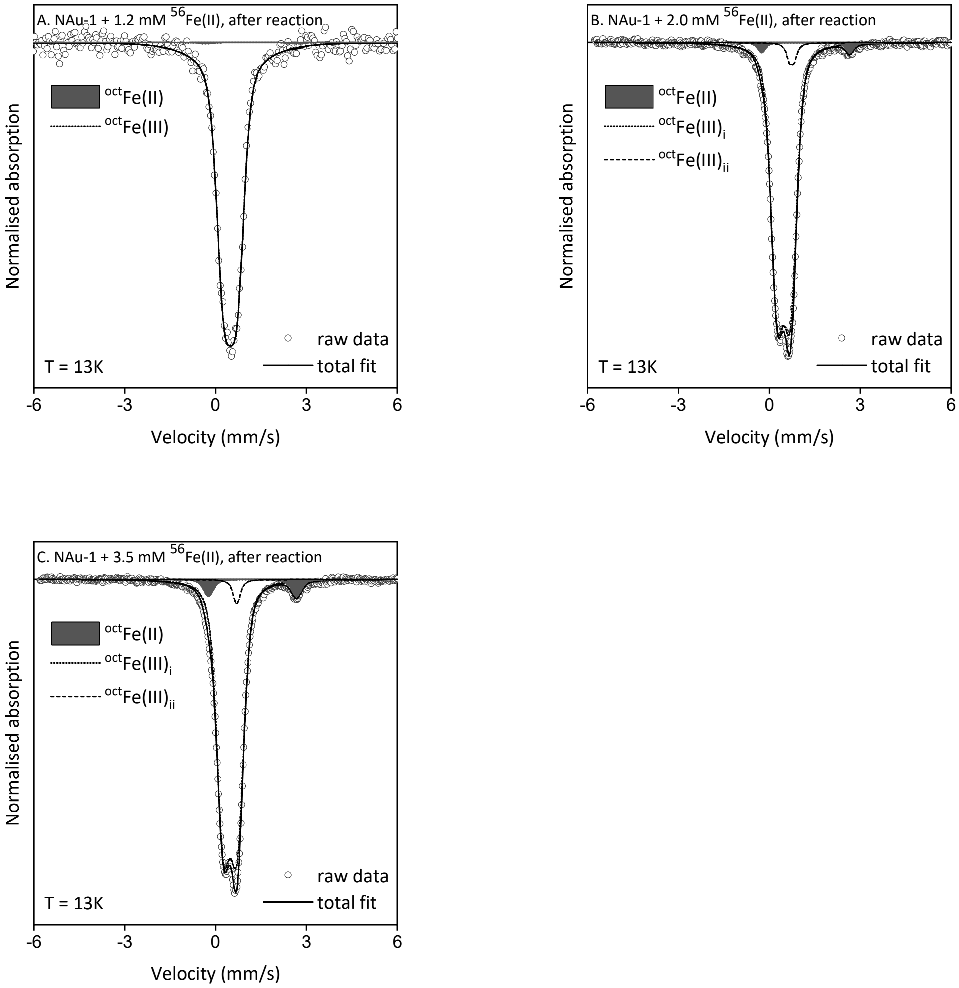

Figure S 6: Mössbauer spectra measured after the reaction with 2 AcNB. of (a) 1.2 mM ^{56}Fe(II) + NAu-1 (0.2 % Fe(II)/Fe(tot)), (b) 2.0 mM ^{56}Fe(II) + NAu-1 (2.0 % Fe(II)/Fe(tot)), and (c) 3.5 mM ^{56}Fe(II) + NAu-1 (4.7 % Fe(II)/Fe(tot)).

Table S 1: Values of the Mössbauer hyperfine parameters Centre Shift (CS) and Quadrupole Split (QS) for ^{56}Fe(II)-reduced NAu-1, measured at 13 K and fitted using a Voigt-based line shape [5].

NAu-1 sample	χ^2	Site	CS (mm/s)[a]	QS (mm/s)[b]	Area (%)[c]
		**Before reaction with 2AcNB**			
+ 1.2 mM ^{56}Fe(II)	0.63	oct Fe(III)i	0.47	0.45	93.5 ±1.2
		oct Fe(III)ii	0.73	0.16	2.9 ± 1.1
		oct Fe(II)	1.20	2.97	3.58 ± 0.54
+ 2.0 mM ^{56}Fe(II)	0.62	oct Fe(III)i	0.48	0.50	91.57 ±0.81
		oct Fe(III)ii	0.73	0.08	1.86 ± 0.64
		oct Fe(II)	1.21	2.90	5.57 ± 0.56
+ 3.5 mM ^{56}Fe(II)	0.73	oct Fe(III)i	0.48	0.47	89.90 ±0.88
		oct Fe(III)ii	0.70	0.18	2.10 ±0.71
		oct Fe(II)	1.20	2.85	8.00 ± 0.60
		**After reaction with 2AcNB**			
+ 1.2 mM ^{56}Fe(II)	0.59	oct Fe(III)i	0.49	0.35	99.8 ±1.9
		oct Fe(II)	1.20	3.00	0.2 ± 1.9
+ 2.0 mM ^{56}Fe(II)	0.87	oct Fe(III)i	0.48	0.44	93.20 ±0.61
		oct Fe(III)ii	0.74	0.15	3.38 ± 0.54
		oct Fe(II)	1.20	2.90	2.02 ± 0.32
+ 3.5 mM ^{56}Fe(II)	0.97	oct Fe(III)i	0.48	0.46	92.31 ±0.34
		oct Fe(III)ii	0.70	0.10	2.93 ±0.22
		oct Fe(II)	1.22	2.90	4.76 ± 0.28

[a] Center shift relative to α-Fe(0). [b] ± values indicate standard deviation of QS from the Gaussian distribution of the QS parameter used in the model. [c] ± values indicate standard deviation due to uncertainty.

S8 Input and output parameters of the two-site kinetic model

Table S 2: Rate constants of the reactive Fe(II) site(s), k_A, k_B (log values) and the initial concentration of highly reactive Fe(II) sites, $[\mathrm{Fe(II)_A}]$, for the reduction of probe compound 2-acetylnitrobenzene (2AcNB) by dithionite- and Fe(II)-reduced clay mineral NAu-1 containing different Fe(II)/Fe(total) ratios and total clay mineral Fe(II) concentrations. Clay mineral Fe reduction potential values, E_H, were calculated for each Fe(II)/Fe(total) ratio based on the modified Nernst equation and parameters provided in ref [6], both for the native (E_H^{nat}) and fully reduced (E_H^{red}) clay mineral NAu–1. All values given ± standard deviation.

**NAu-1 Fe(II)/Fe(total)** (%)[a]	**NAu-1 [Fe(II)]** (mM)[b]	$\mathbf{E_H^{nat}}$ (V)	$\mathbf{E_H^{red}}$ (V)	**log $\mathbf{k_A}$** [k/M^{-1}h^{-1}]	**log $\mathbf{k_B}$** [k/M^{-1}h^{-1}]	$\mathbf{[Fe(II)_A]}$ (mM)
**Dithionite-reduced NAu-1**						
3.5	0.32	-0.29±0.01	2.34	-	-	-
5	0.45	-0.30±0.01	2.15	1.54±0.09	-1.11±0.13	0.11±0.04
10	0.81	-0.34±0.01	1.77	2.89±0.14	-0.26±0.07	0.10±0.03
13	1.17	-0.36±0.01	1.61	2.88±0.17	0.07±0.1	0.12±0.06
30	2.70	-0.41±0.01	1.07	3.72±0.07	-0.38[c]	0.35±0.03
48	4.41	-0.45±0.01	0.68	3.38±0.27	-0.4[c]	1.11±0.07
75	6.75	-0.50±0.01	0.08	3.70±0.09	-0.45[c]	0.34±0.06
91	8.19	-0.56±0.01	-0.55	3.53±0.07	-0.47[c]	0.40±0.04
**Fe(II)-reduced NAu-1**						
3.5	0.32	-0.29±0.01	2.34	-[d]	0.71±0.04[d]	-[e]
5.5	0.50	-0.31±0.01	2.10	2.31±0.11	0.75±0.04	0.10±0.06
8	0.72	-0.33±0.01	1.89	2.78±0.21	0.76±0.06	0.10±0.09

[a] Relative area of the Fe(II) doublet in the Mössbauer spectra of dithionite-reduced NAu-1 (Table S4) or ^{56}Fe(II)-reacted NAu-1 (Table S1). [b] Calculated using the concentration of clay mineral Fe (0.45 mM/g) and the reduction extent of NAu-1 (Fe(II)/Fe(total) ratio). [c] The uncertainty of log k_B values increased significantly at Fe(II)/Fe(total) ratios exceeding 30% and are thus not shown. We attribute this to the kinetics being dominated by the highly reactive Fe(II) sites at higher reduction extents. Regardless of the uncertainty associated with the absolute value of log k_B, low reactivity Fe(II) sites need to be invoked to correctly describe the observed 2AcNB reduction kinetics and contribute more to the overall clay mineral Fe(II) reactivity at lower reduction extents. [d] 2AcNB reduction kinetics were described by a second-order kinetic rate law given in eq 3, indicative of only one reactive Fe(II) site and more alike those of the less reactive Fe(II) sites in eq 1. [e] The kinetic model used here (eq 3) only includes one reactive Fe(II) site and thus $[\mathrm{Fe(II)_A}]$ is equal to the total Fe(II) concentration in the reactor.

S9 NAu-1 Reduction Potential

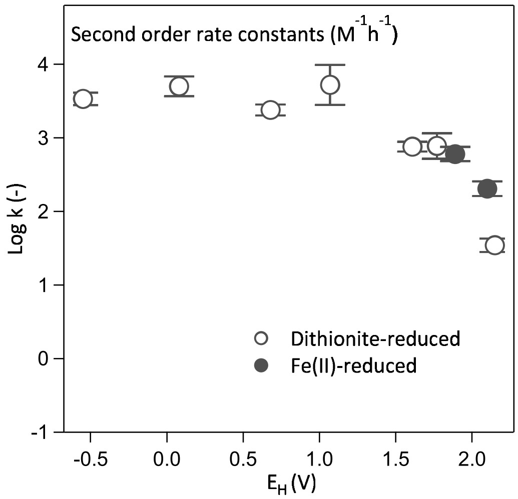

Figure S 7: Second-order rate constants (log (k_A/M^{-1}h^{-1})) of the highly reactive Fe(II) sites in NAu-1 plotted against the NAu-1 reduction potential calculated from ref [6] for reduced NAu-1 (E$_H^{red}$, listed in Table S2).

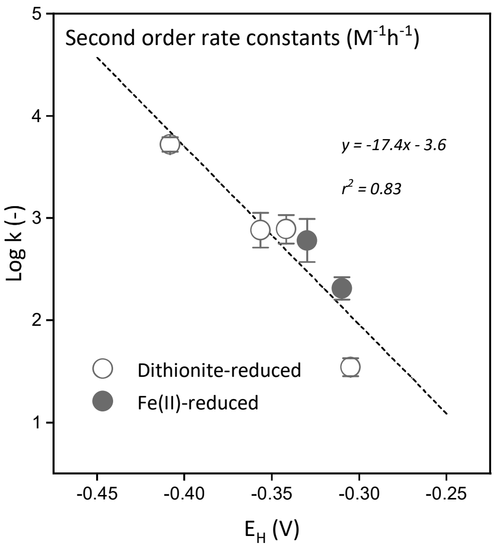

Figure S 8: Second-order rate constants (log (k_A/M^{-1}h^{-1})) of the highly reactive Fe(II) sites in NAu-1 plotted against the NAu-1 reduction potential calculated from ref [6] for native NAu-1 (E$_H^{nat}$, listed in Table S2), for Fe(II)/Fe(total) ratios comparable to those in ref [7] ($\leq$30%).

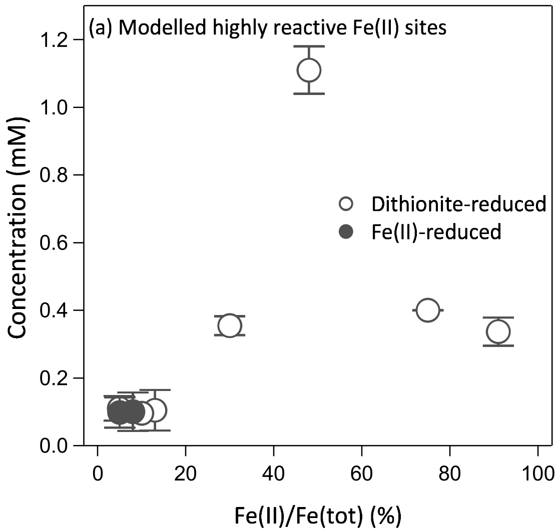

Figure S 9: Initial concentrations of highly reactive Fe(II) sites ($[\mathrm{Fe(II)_A}]$) in dithionite- (open circles) and Fe(II)-reduced NAu-1 (filled circles) as a function of clay mineral Fe(II)/Fe(total) ratio, which varied between 3.5 and 91%. Error bars indicate ± standard deviation.

S10 Electron Balance Calculations

Table S 3: Electron balance calculations for 2-acetylnitrobenzene (2AcNB) degradation experiments with Fe(II)-reduced NAu-1.

	Before 2AcNB addition					After reaction with 2AcNB			
**NAu-1 reduction extent**[a]	**aqueous Fe(II) initial**	**aqueous Fe(II) final**	**Fe(II) taken up total**[b]	**Fe(II) taken up remaining**[c]	**NAu-1 Fe reduced**[d]	**aqueous Fe(II) final**	**Fe(II) taken up additionally**[e]	**NAu-1 Fe(II) remaining**	
Fe(II)/Fe(total)	mM	mM	mM	mM	mM	mM	mM	Fe(II)/Fe(total)[a]	mM[d]
3.5%	1.2	0.67	0.53	0.21	0.32	0.23	0.24	0.2%	0.02
5.5%	2.0	1.14	0.86	0.45	0.41	0.73	0.41	2.0%	0.15
8.0%	3.5	2.03	1.47	0.75	0.72	1.25	0.78	4.7%	0.42

[a] Relative area of the Fe(II) doublet in the Mössbauer spectra of ^{56}Fe(II)-reacted NAu-1. Data from Table S1. [b] Calculated from the difference of initial and final aqueous Fe(II) concentration. [c] Calculated as the difference between the total Fe(II) taken up and Fe(II) oxidised, which is equal to the NAu-1 Fe reduced during interfacial electron transfer from added Fe(II). [d] Calculated using the concentration of clay mineral Fe (0.45 mM/g) and the Fe(II)/Fe(total) ratio of NAu-1, as determined in the Mössbauer spectra of ^{56}Fe(II)-reacted NAu-1 (Table S1). [e] Calculated as the difference between final aqueous Fe(II) concentrations before and after reaction with 2AcNB.

S11 Calculation of reduction extent at which proton uptake dominates over cation sorption

Drits and Manceau (2000) [8] proposed a model relating nontronite reduction extent ($\frac{m}{m_0}$), cation sorption (p), proton uptake (n_i) and a constant (K_0) as shown in Equation S1. Proton uptake dominates over cation sorption if $\frac{n_i}{p} > 1$.

$$\frac{n_i}{p} = K_0 \frac{m}{m_0} \tag{S1}$$

Where K_0 is dependent on the cation exchange capacity (CEC) and structural Fe content (m_{tot}) of the nontronite calculated as shown in Equation S2:

$$K_0 = CEC(9.32 - 1.06m_{tot} + 0.02{m_{tot}}^2) \tag{S2}$$

We used the following values in our calculations:

- CEC of NAu-1: 1.17 mmol/g (from ref [9])
- NAu-1 m_{tot}: 3.76 mmol/g (measured through HF digestion)
- Calculated K_0: 6.57
- Calculated reduction extent where proton uptake dominates over cation sorption: 15.2 %

S12 Mössbauer spectra of dithionite-reduced NAu-1

As detailed in Table S4, the spectra generally show an octahedral Fe(III) doublet comprising most of the Fe(III) spectral area (black dots) with parameters centre shift (CS) = 0.38 - 0.49, quadrupole splitting (QS) = 0.45 - 0.68 and a second, sometimes absent, smaller octahedral Fe(III) doublet (black dashes) with parameters CS = 0.61 - 0.75, QS = 0.08 - 0.18. At reduction extents ≤30 % Fe(II)/Fe(tot), Fe(II) was fit as a doublet (filled blue) with the parameters CS = 1.20 - 1.23, QS = 2.8 - 2.95. Above 30 % Fe(II)/Fe(tot), two Fe(II) doublets (filled blue and patterned blue) were required to fit the spectra with the parameters CS = 1.24 - 1.29, QS = 2.02 - 3.05, and CS = 1.14 - 1.27, QS = 2.74 - 2.84. Above 30 % Fe(II)/Fe(tot), spectra measured at 13 K exhibited partial magnetic ordering, which is included in the fit as grey lines showing broad, poorly resolved features.

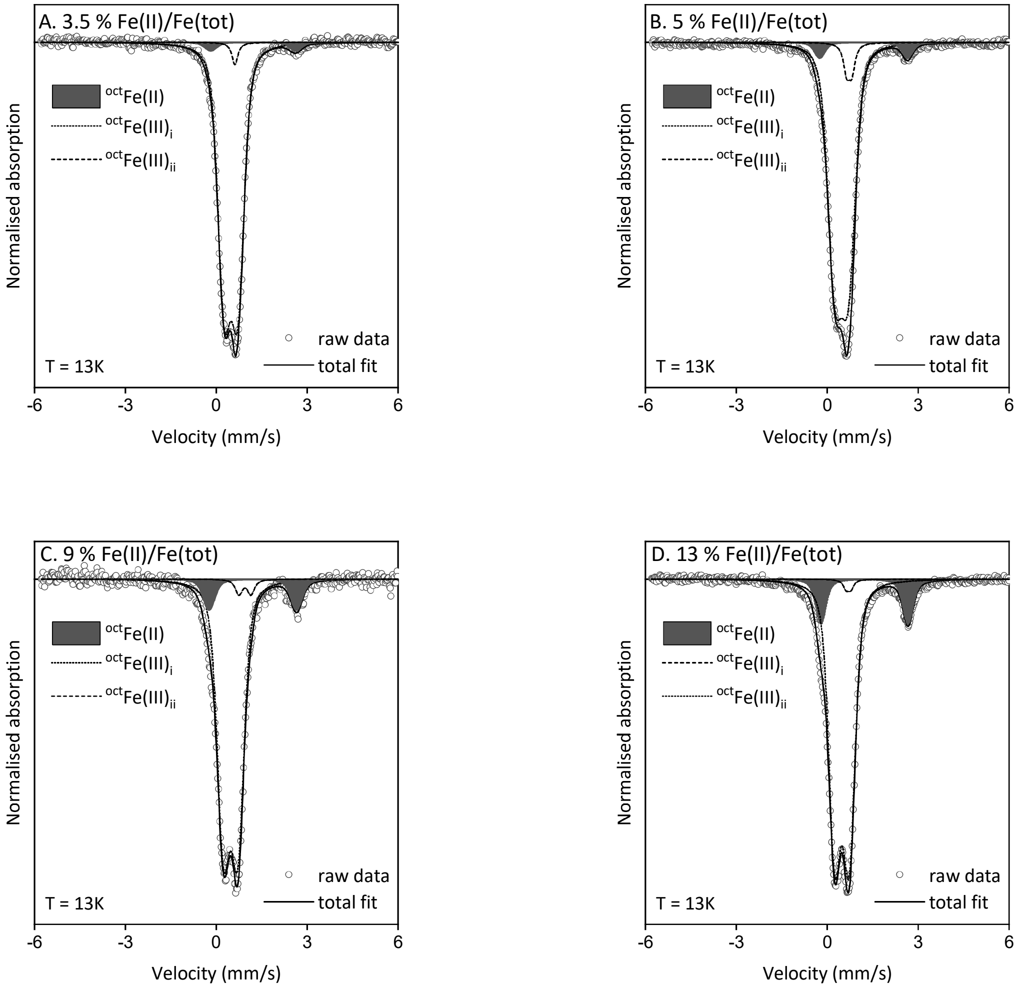

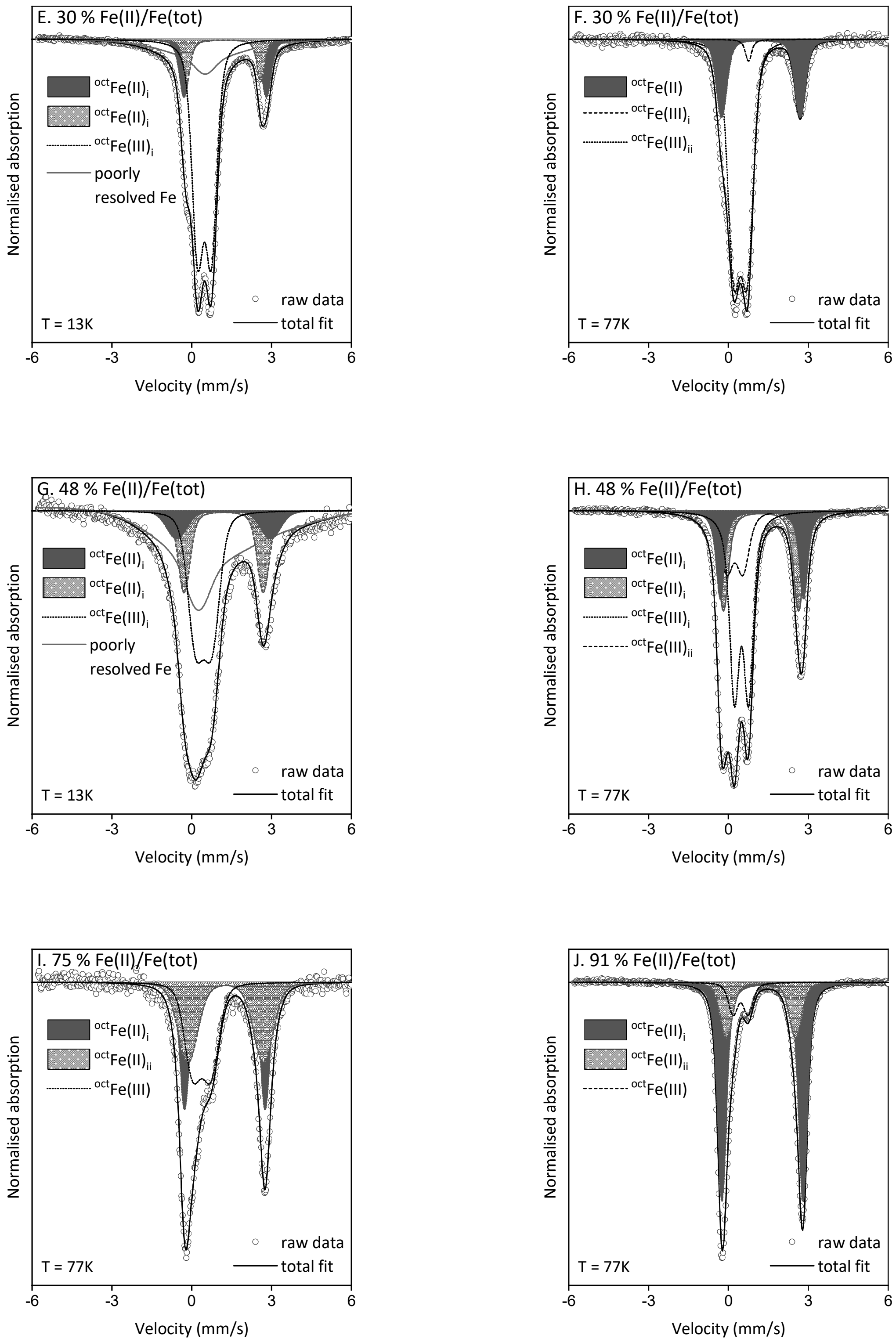

Figure S 10: (a) 3.5% dithionite-reduced NAu-1 measured at 13 K, (b) 5% dithionite-reduced NAu-1 measured at 13 K, (c) 9% dithionite-reduced NAu-1 measured at 13 K, (d) 13% dithionite-reduced NAu-1 measured at 13 K, (e) 30% dithionite-reduced NAu-1 measured at 13 K, partial magnetic ordering is evident as broadening, comprising a poorly resolved Fe ordered phase, (f) 30% dithionite-reduced NAu-1 measured at 77 K showing no ordering, (g) 48% dithionite-reduced NAu-1 measured at 13 K, partial magnetic ordering is evident as broadening, comprising a poorly resolved Fe ordered phase, (h) 48% dithionite-reduced NAu-1 measured at 77 K showing no ordering, (i) 75% dithionite-reduced NAu-1 measured at 77 K, and (j) 91% dithionite-reduced NAu-1 measured at 77 K.

Table S 4: Values of the Mössbauer hyperfine parameters Centre Shift (CS), Quadrupole Split (QS) and Hyperfine magnetic field (H) for dithionite-reduced NAu-1, measured at 13 K or 77 K and fitted using a Voigt-based line shape [5].

**NAu-1 sample**	χ^2	**Site**	**CS (mm/s)**[a]	**QS (mm/s)**[b]	**H (T)**	**Area (%)**[c]
3.5% reduced	0.71	oct Fe(III)i	0.49	0.45	-	94.32 ±0.69
13 K		oct Fe(III)ii	0.61	0.05*	-	2.16 ±0.50
		oct Fe(II)	1.22	2.8*	-	3.52 ±0.51
5% reduced	0.65	oct Fe(III)i	0.47	0.48	-	88.92 ±0.70
13 K		oct Fe(III)ii	0.73	0.17	-	6.00 ±0.61
		oct Fe(II)	1.20	2.90	-	5.08 ±0.41
9% reduced	0.71	oct Fe(III)i	0.47	0.48	-	87.8 ±1.2
13 K		oct Fe(III)ii	0.75	0.4*	-	3.22 ±0.91
		oct Fe(II)	1.21	2.89	-	9.01 ±0.41
13% reduced	1.75	oct Fe(III)i	0.49	0.46	-	80.2 ±0.0
13 K		oct Fe(III)ii	0.43	0.62	-	6.8 ± 0.2
		oct Fe(II)	1.23	2.89	-	13.0 ± 0.0
30% reduced	3.03	oct Fe(III)i	0.48	0.52	-	57.12 ±0.60
13 K		oct Fe(II)i	1.26	3.09	-	13.92 ± 0.53
		oct Fe(II)ii	1.20	2.77	-	8.4 ± 2.1
		Poorly resolved Fe[d]	0.5*	0	9.89 ± 53	20.60 ± 0.80
30% reduced	1.11	oct Fe(III)i	0.45	0.51	-	68.12 ±0.64
77 K		oct Fe(III)ii	0.75	0.09	-	2.09 ± 0.56
		oct Fe(II)	1.22	2.95	-	29.70 ± 50
48% reduced	0.72	oct Fe(III)i	0.45	0.61	-	28.9 ±4.2
13 K		oct Fe(II)i	1.20	3.52	-	7.9 ± 9.5
		oct Fe(II)ii	1.20	2.97	-	15.1 ± 8.2
		Poorly resolved Fe[d]	0.98	0.74	12± 41	48.1 ± 6.9
48% reduced	1.17	oct Fe(III)i	0.46	0.55	-	47.6 ±0.7
77 K		Fe(III)ii	0.1	0.49	-	4.0 ± 0.1
		oct Fe(II)i	1.26	3.05	-	36.0 ± 0.7
		oct Fe(II)ii	1.14	2.81	-	12.4 ± 0.8
75% reduced	0.61	oct Fe(III)	0.38	0.68	-	25.3 ±3.0
77 K		oct Fe(II)i	1.24	3.03	-	38.3 ± 4.8
		oct Fe(II)ii	1.27	2.84	-	36.3 ± 4.6
91% reduced	1.67	oct Fe(III)	0.48	0.55	-	8.7 ±0.1
77 K		oct Fe(II)i	1.29	3.02	-	63.2 ± 0.6
		oct Fe(II)ii	1.21	2.74	-	28.1 ± 0.1

[a] Center shift relative to α-Fe(0). [b] ± values indicate standard deviation of QS from the Gaussian distribution of the QS parameter used in the model. [c] ± values indicate standard deviation due to uncertainty.

[d] Large uncertainty is associated with the poorly resolved phases due to their partially ordere nature.

* indicates parameter was fixed during fitting.

S13 Mössbauer spectra of dithionite-reduced NAu-1 at 4K

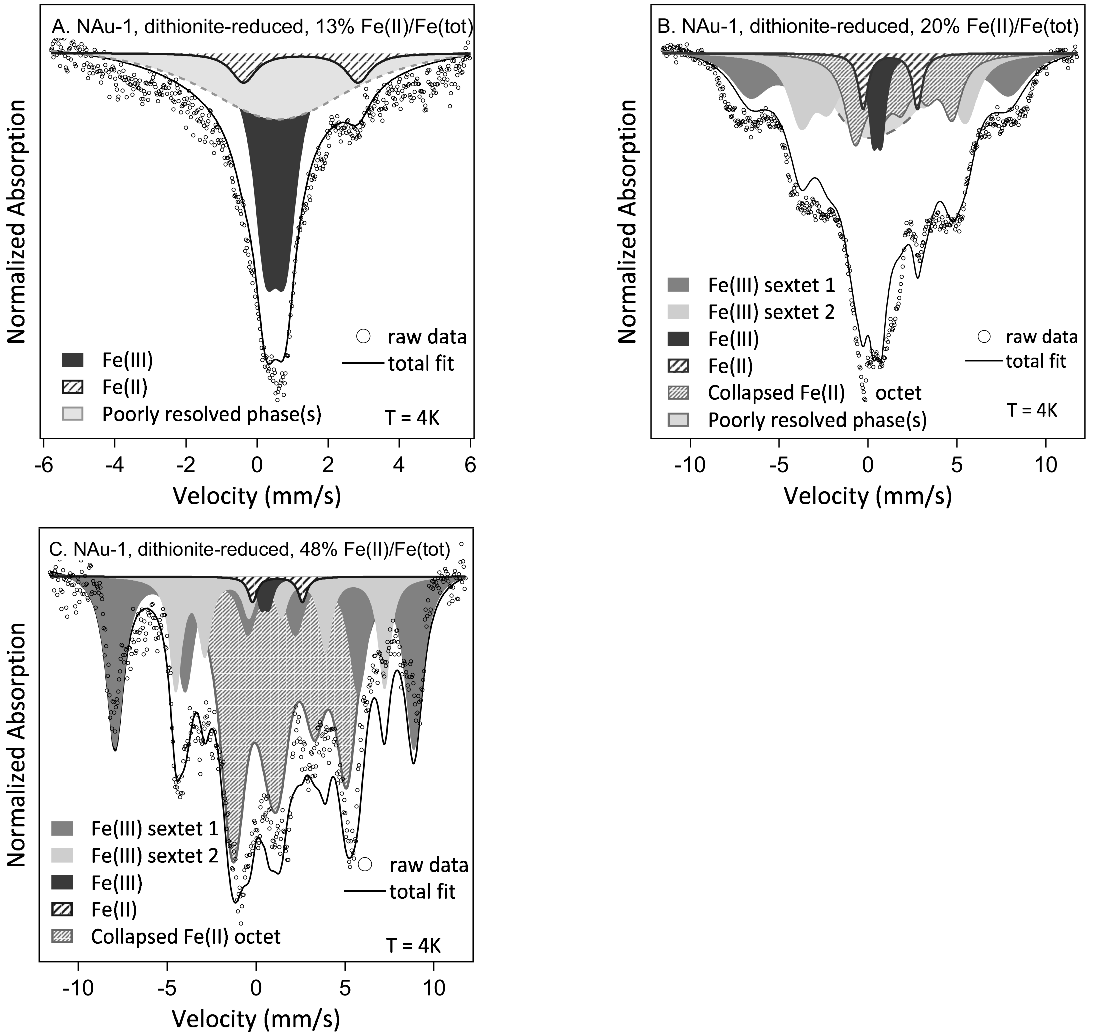

Figure S 11: Mössbauer spectra collected at 4 K of (a) 13% dithionite-reduced NAu-1, (b) 20% dithionite-reduced NAu-1, and (c) 48 % dithionite-reduced NAu-1.

At 13 % Fe(II)/Fe(tot), magnetic ordering is visible in the spectrum as a broad, poorly resolved feature (56.6 % of the spectral area). At 20 % Fe(II)/Fe(tot) and above, two clearly resolved sextets are visible (beige and orange areas in Figure S8B, 47% of total area) and exhibit hyperfine parameters (Centre shift (CS: 0.45-0.65 mm/s), Hyperfine magnetic field (H: 29-45 T), Table S5) consistent with magnetically ordered octahedral Fe(III) in nontronites[10]. Additionally, we found an Fe(II) octet (blue shaded area in Figure S8B, 18% of total area) that is not well resolved (H: 12.2 T, Table S5) but can be fit using parameters previously reported for magnetically ordered Fe(II) in trioctahedral annite and ferrous biotite[11, 12] (Table S6). Two doublets occupy a small spectral area (9%) and comprise Fe(II) and Fe(III) species (dark blue shaded and red areas, respectively) that have not magnetically ordered. Similarly, the broad, poorly resolved feature similar to that observed in the 13% reduced sample (Figure

S8A) comprises 26% of the spectral area (grey area in Figure S8B). At higher Fe reduction extent, the poorly resolved, broad component is no longer present and identical components as found at 20% Fe(II)/Fe(total) were present in the Mössbauer spectra, with the dominance of the collapsed Fe(II) octet increased with increasing reduction extent, occupying 87% of the spectral area at 91 % Fe(II)/Fe(tot) (Figure 3b (main text), Table S5).

Table S 5: Mössbauer parameters for reduced NAu-1, measured at 4 K using a variable line width fitting method (Full Static Hamiltonian).[13]

	**CS (mm/s)**	**H (T)**	$\mathbf{e^2qQ/2}$ **(mm/s)**[a]	η **(-)**[b]	θ **(-)**[c]	**Area(%)**
			13% dithionite-reduced NAu-1			
Fe(III) doublet	0.52	-	0.54	0	0	36.8
Fe(II) doublet	1.23	-	3.22	0	0	6.6
Partially resolved phase(s)	0.6	2.2	0.5	0	0	56.6
			20% dithionite-reduced NAu-1			
Fe(III) $sextet_1$	0.45	45.1	0.38	0	0	24.4
Fe(III) $sextet_2$	0.64	28.6	0.44	0	0	22.7
Fe(III) doublet	0.52	-	0.45	0	0	4.7
Fe(II) doublet	1.25	-	3.05	0	0	3.9
Collapsed Fe(II) octet	1.66	12.2	-2.9	0.2	90	18.4
Partially resolved phase(s)	0.5	10	0.45	0	0	25.8
			48% dithionite-reduced NAu-1			
Fe(III) $sextet_1$	0.67	52.2	-0.4	0	0	34.1
Fe(III) $sextet_2$	0.93	36.3	0.84	0	0	14.4
Fe(III) doublet	0.5	-	0.45	0	0	1.2
Fe(II) doublet	1.2	-	2.8	0	0	1.3
Collapsed Fe(II) octet	1.47	16.4	-2.77	0.22	90	49.0
			91% dithionite-reduced NAu-1			
Fe(III) $sextet_1$	0.52	54.7	0.01	0	0	1.6
Fe(III) $sextet_2$	0.91	30.1	1.4	0	0	4.1
Fe(III) doublet	0.6	-	0.45	0	0	2.9
Fe(II) doublet	1.45	-	2.91	0	0	4.3
Collapsed Fe(II) octet	1.44	17.3	-2.6	0.2	90	87.1

[a] Quadrupole splitting in paramagnetic state or quadrupole shift.
[b] Asymmetry parameter. [c] Polar angle between the electric field gradient (EFG) axis of symmetry with hyperfine field.

S14 Mössbauer parameters for Fe-bearing phyllosilicates from literature

Table S 6: Mössbauer parameters gathered from literature for trioctahedral and dioctahedral phyllosilicates. All samples were measured at 4.2 K.

Mineral	Component	CS (mm/s)	QS (mm/s)	H (T)	η (-)	$\theta(^{\circ})$	Reference
Ferrous	Fe(III) sextet 1	*	*	53	*	*	[12]
biotite	Fe(III) sextet 2	*	*	48	*	*	
(trioctahedral)	Fe(II) octet	*	-2.75	15	0.2	90	
Nontronite	Fe(III) sextet	*	*	49	*	*	[12]
Nontronite	Fe(III) sextet	*	*	34 - 48	*	*	[10]

* Data not provided.

S15 Mössbauer spectra of ^{57}Fe(II)-reduced NAu-1

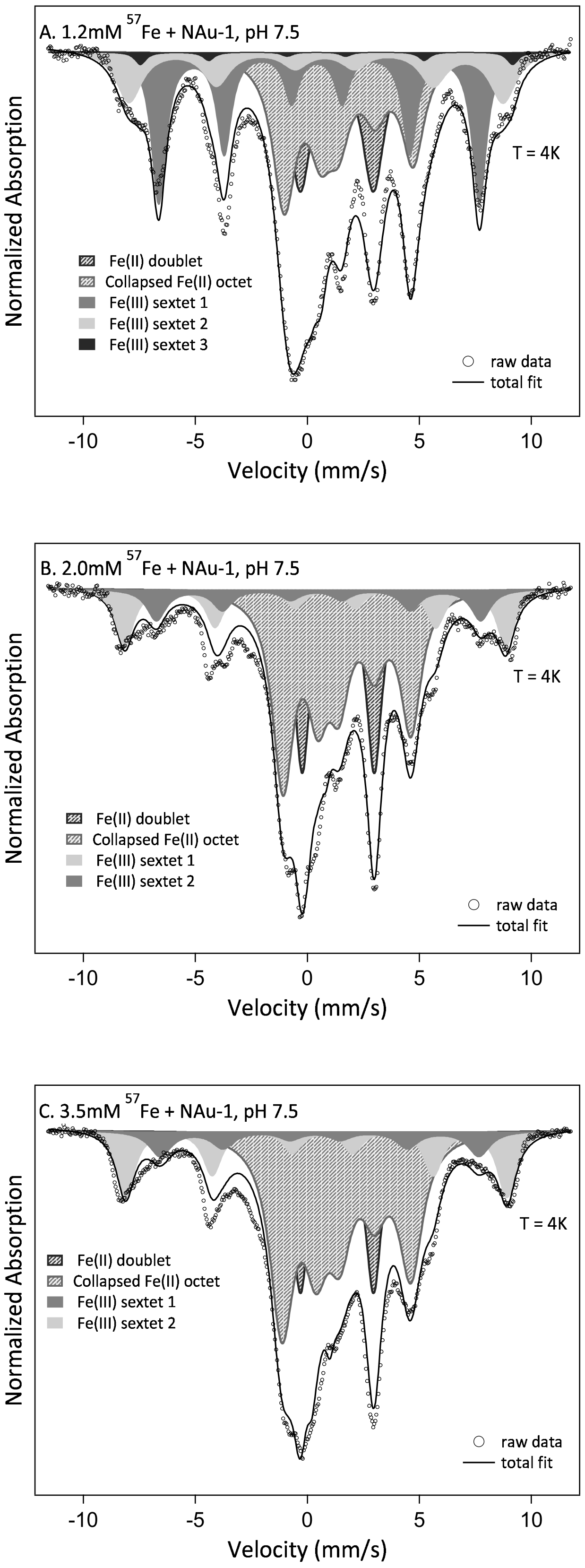

Figure S 12: Mössbauer spectra of (a) 1.2mM, (b) 2.0mM and (c) 3.5mM ^{57}Fe(II) + NAu-1, at pH 7.5, measured at 4K.

Table S 7: Mössbauer parameters for $^{57}Fe(II)$ + NAu-1, measured at 4 K using a variable line width fitting method (Full Static Hamiltonian).

	**CS (mm/s)**	**H (T)**	**$e^2qQ/2$ (mm/s)**	**η (-)**	**θ (-)**	**Area(%)**
			1.2 mM $^{57}Fe(II)$ + NAu-1			
Fe(III) sextet 1	0.64	52.0	-0.40	0	0	14.3
Fe(III) sextet 2	0.53	44.5	-0.01	0	0	33.5
Fe(II) doublet	1.46	-	3.01	0	0	15.0
Collapsed Fe(II) octet	1.40	12.7	-2.73	0	90	37.3
			2.0 mM $^{57}Fe(II)$ + NAu-1			
Fe(III) sextet 1	0.52	53.4	-0.27	0	0	11.4
Fe(III) sextet 2	0.55	45.7	0.10	0	0	17.6
Fe(II) doublet	1.41	-	3.16	0	0	25.5
Collapsed Fe(II) octet	1.35	13.1	-2.83	0	90	45.4
			3.5 mM $^{57}Fe(II)$ + NAu-1			
Fe(III) sextet 1	0.50	53.4	-0.26	0	0	10.5
Fe(III) sextet 2	0.54	48.1	-0.09	0	0	13.2
Fe(II) doublet	1.40	-	3.12	0	0	24.2
Collapsed Fe(II) octet	1.33	12.8	-3.03	0	90	52.2

S16 Reactivity of sorbed Fe(II)

Table S 8: Rate constants for NAC reduction by Fe(II)-silica, alumina and lepidocrocite from previous studies. Note these values have not been adjusted for Fe loadings or surface area.

**Phase**	**Mineral load (g/l)**	**Fe(II) conc (mM)**	**pH**	**log k_{obs}**	**ref**
Silica	10	0.31 sorbed<br>0.81 total	7.5	1.1E-04	[14]
Alumina	10	0.69 sorbed<br>1.19 total	7.5	3.35E-05	[14]
Lepidocrocite$_1$	2.7	$0.5_{(aq)}$	7.2	0.49	[15]
Lepidocrocite$_2$	2.7	$1.0_{(aq)}$	7.2	0.92	[15]
Lepidocrocite$_3$	10	$1.0_{(aq)}$	7.2	1.03	[15]
Lepidocrocite$_4$	1.42	$1.0_{(aq)}$	7.2	0.12	[16]

References

[1] Bingjie Shi, Kai Liu, Lingling Wu, Weiqiang Li, Christina M Smeaton, Brian L Beard, Clark M Johnson, Eric E Roden, and Philippe Van Cappellen. Iron isotope fractionations reveal a finite bioavailable fe pool for structural Fe(III) reduction in nontronite. *Environmental Science & Technology*, 50(16):8661–8669, 2016.

[2] Peter Komadel, Jana Madejova, and Joseph W Stucki. Reduction and reoxidation of nontronite: Questions of reversibility. *Clays and Clay Minerals*, 43(1):105–110, 1995.

[3] Michael V Schaefer, Christopher A Gorski, and Michelle M Scherer. Spectroscopic evidence for interfacial Fe(II)-Fe(III) electron transfer in a clay mineral. *Environmental Science & Technology*, 45(2):540–545, 2011.

[4] Fubo Luan, Yan Liu, Aron M Griffin, Christopher A Gorski, and William D Burgos. Iron (iii)-bearing clay minerals enhance bioreduction of nitrobenzene by shewanella putrefaciens cn32. *Environmental science & technology*, 49(3):1418–1426, 2015.

[5] DG Rancourt and JY Ping. Voigt-based methods for arbitrary-shape static hyperfine parameter distributions in Mössbauer spectroscopy. *Nuclear Instruments and Methods in Physics Research Section B: Beam Interactions with Materials and Atoms*, 58(1):85–97, 1991.

[6] Christopher A Gorski, Laura E Klüpfel, Andreas Voegelin, Michael Sander, and Thomas B Hofstetter. Redox properties of structural Fe in clay minerals: 3. relationships between smectite redox and structural properties. *Environmental Science & Technology*, 47(23):13477–13485, 2013.

[7] Fubo Luan, Christopher A Gorski, and William D Burgos. Linear free energy relationships for the biotic and abiotic reduction of nitroaromatic compounds. *Environmental Science & Technology*, 49(6):3557–3565, 2015.

[8] VA Drits and A Manceau. A model for the mechanism of Fe^{3+} to Fe^{2+} reduction in dioctahedral smectites. *Clays and Clay Minerals*, 48(2):185–195, 2000.

[9] Jebril Hadi, Christophe Tournassat, Ioannis Ignatiadis, Jean Marc Greneche, and Laurent Charlet. Modelling CEC variations versus structural iron reduction levels in dioctahedral smectites. existing approaches, new data and model refinements. *Journal of colloid and interface science*, 407:397–409, 2013.

[10] Fabien Baron, Sabine Petit, Martin Pentrák, Alain Decarreau, and Joseph W Stucki. Revisiting the nontronite Mössbauer spectra. *American Mineralogist: Journal of Earth and Planetary Materials*, 102(7):1501–1515, 2017.

[11] DG Rancourt, IAD Christie, G Lamarche, I Swainson, and S Flandrois. Magnetism of synthetic and natural annite mica: ground state and nature of excitations in an exchange-wise two-dimensional easy-plane ferromagnet with disorder. *Journal of Magnetism and Magnetic Materials*, 138(1-2):31–44, 1994.

[12] O Ballet and JMD Coey. Magnetic properties of sheet silicates; 2:1 layer minerals. *Physics and Chemistry of Minerals*, 8(5):218–229, 1982.

[13] Nikolaus Blaes, Harald Fischer, and Ulrich Gonser. Analytical expression for the Mössbauer line shape of ^{57}Fe in the presence of mixed hyperfine interactions. *Nuclear Instruments and Methods in Physics Research Section B: Beam Interactions with Materials and Atoms*, 9(2):201–208, 1985.

[14] Christopher A Schultz and Timothy J Grundl. pH dependence on reduction rate of 4-cl-nitrobenzene by Fe(II)/montmorillonite systems. *Environmental Science & Technology*, 34(17):3641–3648, 2000.

[15] Dimin Fan, Miranda J Bradley, Adrian W Hinkle, Richard L Johnson, and Paul G Tratnyek. Chemical reactivity probes for assessing abiotic natural attenuation by reducing iron minerals. *Environmental Science & Technology*, 50(4):1868–1876, 2016.

[16] Martin Elsner, René P Schwarzenbach, and Stefan B Haderlein. Reactivity of Fe(II)-bearing minerals toward reductive transformation of organic contaminants. *Environmental Science & Technology*, 38(3):799–807, 2004.
